# A convenient four-component one-pot strategy toward the synthesis of pyrazolo[3,4-*d*]pyrimidines

**DOI:** 10.3762/bjoc.11.229

**Published:** 2015-11-06

**Authors:** Mingxing Liu, Jiarong Li, Hongxin Chai, Kai Zhang, Deli Yang, Qi Zhang, Daxin Shi

**Affiliations:** 1 School of Chemical Engineering and Environment, Beijing Institute of Technology, Beijing, 100081, China

**Keywords:** four-component, one-pot, pyrazolo[3,4-*d*]pyrimidine, sodium alkoxide

## Abstract

An efficient one-pot synthesis of pyrazolo[3,4-*d*]pyrimidine derivatives by the four-component condensation of hydrazines, methylenemalononitriles, aldehydes and alcohols has been developed via two different reaction pathways. The structures of target products were characterized by IR spectroscopy, NMR (^1^H and ^13^C) spectroscopy and HRMS (ESI) spectrometry. The crystal structure of 4-ethoxy-6-(2-nitrophenyl)-1-phenyl-1*H*-pyrazolo[3,4-*d*]pyrimidine was determined by single crystal X-ray diffraction.

## Introduction

Heterocycles containing a pyrimidine ring are extensively present in natural products and are very important because of their biological activity [1–6]. They have shown a wide range of pharmacological potential such as kinase inhibitors [1], antitumor [7–8], anti-inflammatory [9–10], antimicrobial [11–13], pesticides [14], radio protectant [15] and cardiovascular activity [16–17]. For example, ibrutinib, sildenafil, allopurinol and zaleplon are famous pyrazolopyrimidine drugs.

Because of the importance of pyrazolo[3,4-*d*]pyrimidines, many methods for the synthesis of pyrazolo[3,4-*d*]pyrimidines have been explored. Some examples include the condensation of 5-aminopyrazole-4-carbonitrile with amides [18–21], carboxylic acids [22–24], amidines [25–26], nitriles [27–28], ketones [29–30] and halohydrocarbon [31], the cyclization of 5-aminopyrazole-4-carboxamides with amides [32], ureas [33–36], esters [37–39] and acyl chloride [40], and the reaction of aminopyrazoles and amides [41–42].

**Scheme 1:**
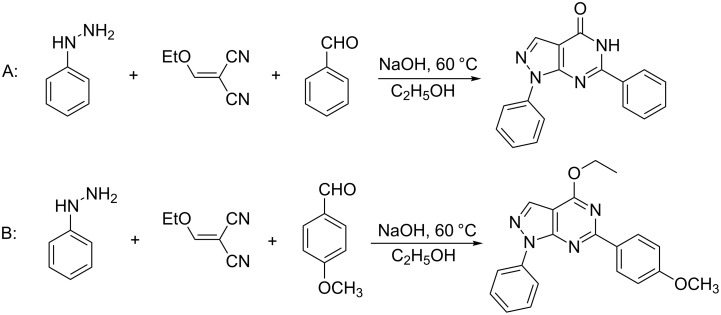
The synthesis of pyrazolo[3,4-*d*]pyrimidines.

In our previous studies, dihydropyrimidinone was synthesized through the condensation of 5-aminopyrazole-4-carbonitrile and ketones [29–30]. 5-Aminopyrazole-4-carbonitrile was prepared from the reaction of ethoxymethylenemalononitrile with phenylhydrazine in a step-wise fashion [43–47]. During the course of previous studies, we envisioned that we could combine these reactions and embarked on designing a strategy toward a one-pot synthesis by combining the three reactants. When benzaldehyde was used as the reactant, the target product was obtained (Scheme 1A). But when benzaldehyde was switched to anisaldehyde, the expected product was not obtained and pyrazolo[3,4-*d*]pyrimidines was isolated (Scheme 1B). Inspired by this phenomenon, we conducted detailed studies and found a new convenient synthesis of pyrazolo[3,4-*d*]pyrimidines. To the best of our knowledge, this is a novel methodology for the synthesis of pyrazolo[3,4-*d*]pyrimidines by the reaction of hydrazines, methylenemalononitriles, aldehydes and alcohols. During the preparation of this manuscript, Liu et al. reported the synthesis of pyrazolo[3,4-*d*]pyrimidines from 5-aminopyrazole-4-carbonitrile [48]. The differences between their and our strategy are that we developed a four-component combined reaction to synthesize pyrazolo[3,4-*d*]pyrimidines, the catalyst that we use is different, the universality of the substrates are very broad and the substrates are more readily available.

## Results and Discussion

Phenylhydrazine, 2-(ethoxymethylene)malononitrile, ethanol and benzaldehyde were selected as the model reactants. The influence of the reaction conditions was studied and the results are summarized in Table 1. No target product was afforded in the presence of an inorganic weak base or without a catalyst (Table 1, entries 1 and 2). Sodium hydroxide could catalyze this reaction, but pyrazolo[3,4-*d*]pyrimidinone **5aa** was obtained instead of pyrazolo[3,4-*d*]pyrimidine **5a** (Table 1, entry 3). This shows that the catalytic properties of sodium hydroxide have some limitations. Fortunately, some strong bases could promote the reaction to produce **5a**, though DBU needed a higher reaction temperature (Table 1, entries 4–6). Taking into account the yield of the reaction, sodium alkoxide was the best choice. The reaction performed in alcohol resulted in the highest yield (Table 1, entries 6–9). The reaction temperature was screened and the appropriate temperature was found to be 60 °C (Table 1, entries 6, 10 and 11). The amount of catalyst had an effect on the reaction and 1.2 equivalents of sodium alkoxide was the most appropriate choice (Table 1, entries 12 and 13). This means that sodium alkoxide is not only a catalyst, but also participates in the reaction.

**Table 1:** Optimization of the reaction conditions^a^.

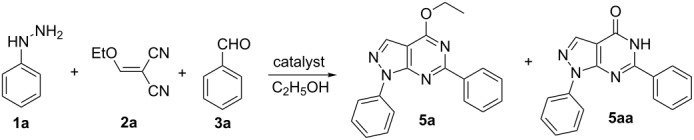				

Entry	Solvent	Cat. (eqiv.)	Temp. (°C)	Yield (%)^b^

1	EtOH	–	60	0
2	EtOH	Na_2_CO_3_ (1.2)	60	0
3	EtOH	NaOH (1.2)	60	82 (**5aa**)
4	EtOH	DBU (1.2)	reflux	42 (**5a**)
5	EtOH	NaH (1.2)	60	62 (**5a**)
6	EtOH	NaOEt (1.2)	60	85 (**5a**)
7	DMSO	NaOEt (1.2)	60	57 (**5a**)
8	toluene	NaOEt (1.2)	60	70 (**5a**)
9	1,4-dioxane	NaOEt (1.2)	60	35 (**5a**)
10	EtOH	NaOEt (1.2)	25	63 (**5a**)
11	EtOH	NaOEt (1.2)	reflux	85 (**5a**)
12	EtOH	NaOEt (0.5)	60	47 (**5a**)
13	EtOH	NaOEt (2.0)	60	85 (**5a**)

^a^Reaction conditions: **1a** (1.2 mmol), **2a** (1.0 mmol), **3a** (1.2 mmol) and catalyst in solvent (15 mL). ^b^Isolated yields.

**Figure 1:**
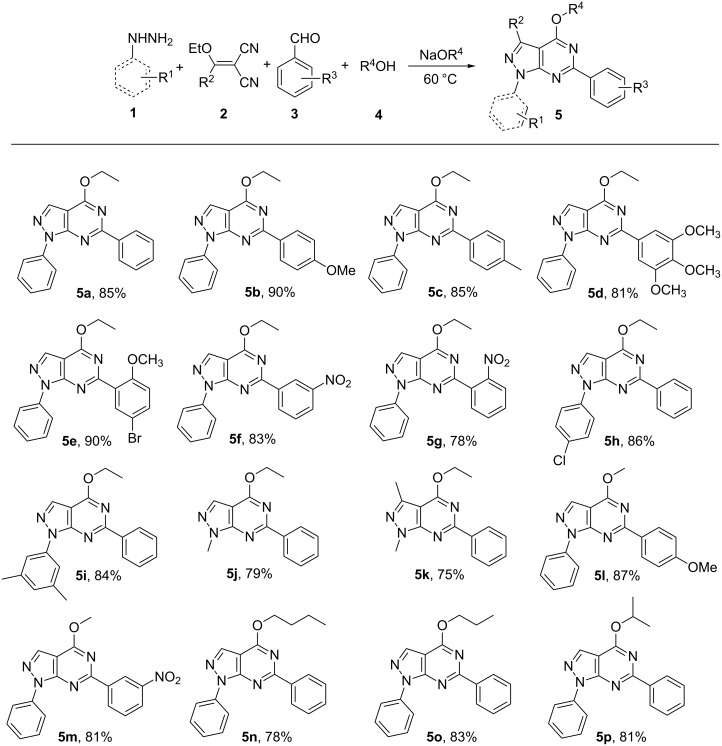
Four-component one-pot synthesis of **5**. Reactions conditions: **1** (1.2 mmol), **2** (1.0 mmol), **3** (1.2 mmol) and **4** (1.2 mmol) in alcohol (15 mL).

A series of hydrazines, methylenemalononitriles, aldehydes and alcohols were investigated under the optimal reaction conditions. As shown in Figure 1, the influence of different aldehydes on the reaction was studied first. The results show that aldehydes with substituents such as *p*-MeO, *p*-Me, 3,4,5-(MeO)_3_, 2-MeO-5-Br, *m*-NO_2_ and *o*-NO_2_ are all compatible under optimal conditions. The corresponding products were obtained in good yield (Figure 1, **5a–g**). Then a set of hydrazines were selected and the target products were obtained. However, the yield of aromatic hydrazines bearing electron-withdrawing groups or electron-donating groups was higher than that of methylhydrazine (Figure 1, **5h–j**). This is possibly due to the electronic effect of the substituents. Though the steric hindrance could affect the reaction, 3-substituted pyrazolo[3,4-*d*]pyrimidine was also obtained in good yield (Figure 1, **5k**). In order to further broaden the scope of this one-pot methodology, a series of alcohols such as methanol, *n*-butanol, *n*-propanol and isopropanol were investigated. The expected products were also obtained in good yield (Figure 1, **5i–p**). This fact revealed the universality and advantages of this method for the synthesis of pyrazolo[3,4-*d*]pyrimidines.

**Scheme 2:**
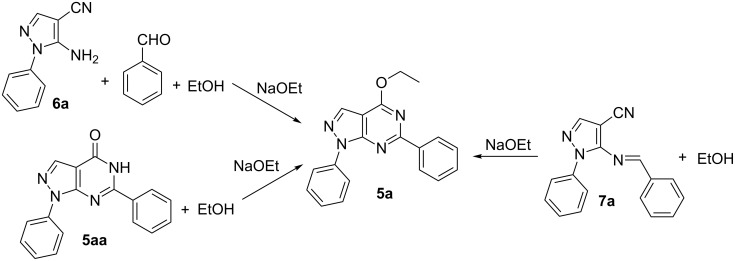
Synthesis of **5a** from different intermediates.

To rationalize the possible reaction mechanism, we successfully separated three intermediates (**6a**, **7a** and **5aa**). 4-Ethoxy-1,6-diphenyl-1*H*-pyrazolo[3,4-*d*]pyrimidine (**5a**) was obtained from the condensation of 5-amino-1-phenyl-1*H*-pyrazole-4-carbonitrile (**6a**) with benzaldehyde and ethanol, the cyclization of (*E*)-5-(benzylideneamino)-1-phenyl-1*H*-pyrazole-4-carbonitrile (**7a**) with ethanol or the reaction of 1,6-diphenyl-1,5-dihydro-4*H*-pyrazolo[3,4-*d*]pyrimidin-4-one (**5aa**) with ethanol, respectively (Scheme 2).

**Scheme 3:**
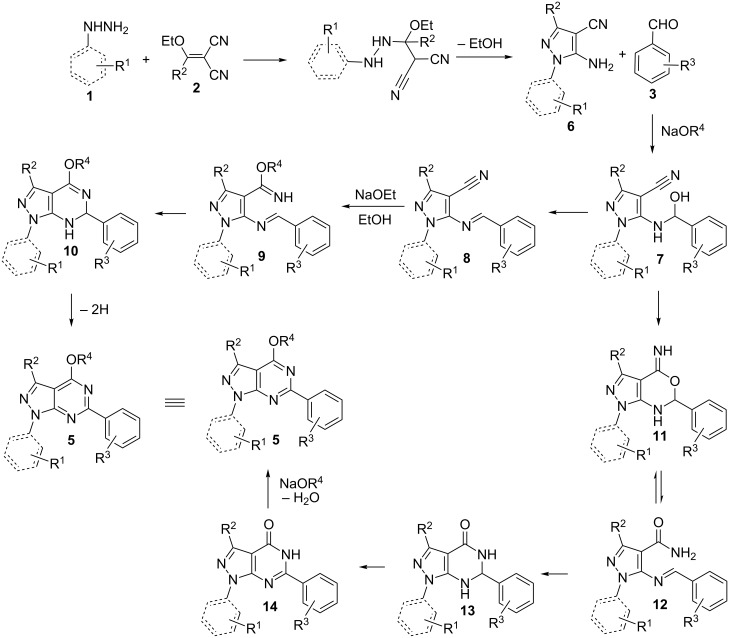
Possible reaction mechanisms for the formation of pyrazolo[3,4-*d*]pyrimidiine.

With those results in hand, two possible reaction mechanisms were proposed and shown in Scheme 3. 5-Aminopyrazole-4-carbonitrile **6** was obtained from the reaction of hydrazine **1** and methylenemalononitrile **2** through nucleophilic addition, cyclization and aromatization. The nucleophilic attack of the amino group of **6** on the carbonyl group of the aldehyde affords **7**. Then **7** provides the target product via two different reaction pathways. The first route is that **7** loses a water molecule to afford the Schiff base **8**. Then **8** undergoes a Pinner reaction and imine **9** is formed, and then **9** turns into **10** through intramolecular cyclization. Finally, **10** is oxidized to give pyrazolo[3,4-*d*]pyrimidine **5**. Another route is that **7** undergoes an intramolecular Pinner reaction to form **11**. Then **11** rearranges to dihydropyrazolo[3,4-*d*]pyrimidin-4-ones **13** via Dimroth rearrangement and **13** is oxidized to provide **14** [49]. Finally, **14** undergoes a nucleophilic addition and loses a water molecule to afford the final product **5**.

**Figure 2:**
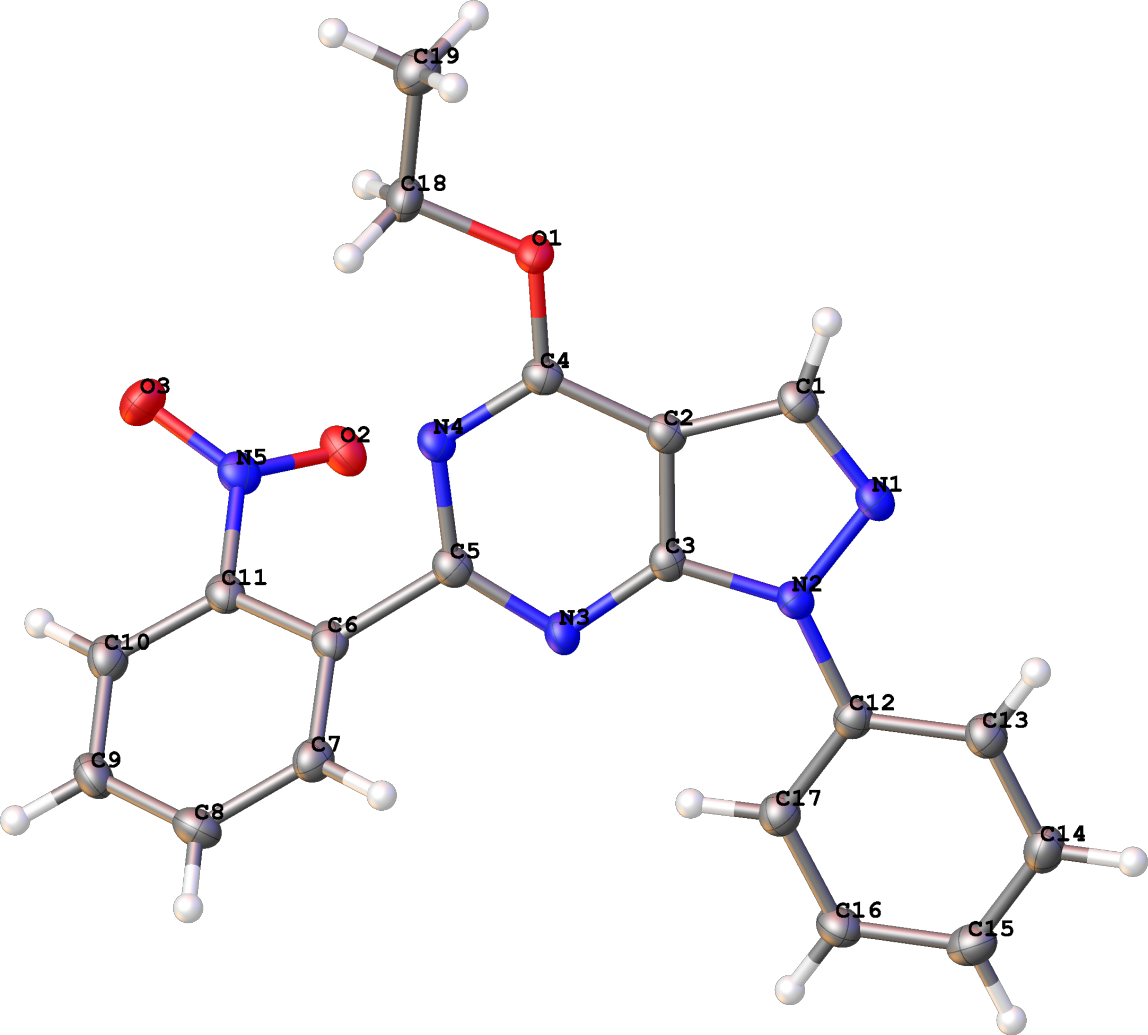
Molecular structure (from X-ray diffraction data) of **5g**.

All products were characterized by IR, ^1^H NMR, ^13^C NMR and HRMS. A final confirmation of the structure of the reaction product **5g** was determined by X-ray diffraction (Figure 2) [50].

## Conclusion

In summary, we have disclosed an efficient one-pot four-component synthesis of pyrazolo[3,4-*d*]pyrimidines. The simplicity of execution, readily available substrates and the potentially important use of the products make this synthetic protocol attractive for academic research and practical applications. Further studies towards the detailed mechanism and synthetic application of this protocol are in progress.

## Supporting Information

File 1 Experimental section and copies of ^1^H and ^13^C NMR spectra of compounds.
