# Supplementary material for: A convenient four-component one-pot strategy toward the synthesis of pyrazolo[3,4-d]pyrimidines
Source: Beilstein J Org Chem. 2015 Nov 6;11:2125–31. doi: 10.3762/bjoc.11.229 (PMC4660965; doi:10.3762/bjoc.11.229)
Supplement: File 1 — Experimental section and copies of 1H and 13C NMR spectra of compounds. [file Beilstein_J_Org_Chem-11-2125-s001.pdf]

Supporting Information

for

A convenient four-component one-pot strategy toward the synthesis of pyrazolo[3,4-*d*]pyrimidines

Mingxing Liu, Jiarong Li, Hongxin Chai, Kai Zhang, Deli Yang, Qi Zhang and Daxin Shi*

Address: School of Chemical Engineering and Environment, Beijing Institute of Technology, Beijing, 100081, China

Email: Daxin Shi - shidaxin@bit.edu.cn.

* Corresponding author

Experimental section and copies of ^{1}H and ^{13}C NMR spectra of compounds

Experimental

Materials and methods

The starting materials are commercially available. Melting points were determined on XT4 microscope melting point apparatus and were uncorrected. Infrared (IR) spectra were recorded on a Perkin Elmer FT-IR spectrophotometer with KBr pellets. ^{1}H and ^{13}C NMR spectra were recorded on a Bruker 400 spectrometer with TMS as the internal standard. Mass spectra were recorded on a Bruker APEX IV using ESI ionization.

General procedure for the synthesis of compound 5. Hydrazine **1** (1.2 mmol), methylenemalononitrile **2** (1.0 mmol) were mixed in alcohol (15 mL), then aldehyde (**3**, 1.2 mmol) and sodium alkoxide (1.2 mmol) were added and the mixture was warmed to 60 °C. The completion of the reaction was monitored by TLC. The reaction solution was cooled to room temperature and diluted with water. The solid was filtered and the filtrate was extracted with acetic ether (3 × 20 mL). Then the organic layer was evaporated under reduced pressure. The solid was combined with filter cake and purified by crystallization from ethanol.

4-Ethoxy-1,6-diphenyl-1*H*-pyrazolo[3,4-*d*]pyrimidine (5a): Yellow solid; m.p. 147-148 ^{o}C; IR (KBr, *v*, cm^{-1}): 3119, 2980, 1593; ^{1}H NMR (400 MHz, DMSO-*d6*) (*δ*, ppm): 8.50-8.48 (m, 2H), 8.45 (s, 1H), 8.31-8.28 (m, 2H), 7.65-7.61 (m, 2H), 7.57-7.56 (m, 3H), 7.43-7.40 (m, 1H), 4.76 (q, *J* = 8.0 Hz, 2H), 1.50 (t, *J* = 8.0 Hz, 3H); ^{13}C NMR (100 MHz, DMSO-d_6) (*δ*, ppm): 163.7, 161.9, 155.8, 139.1, 137.3, 133.7, 131.7, 129.9, 129.8, 129.2, 128.9, 127.1, 121.3, 102.8, 100.0, 63.6, 14.8; HRMS (ESI): calcd. For $C_{19}H_{16}N_4OH$ $[M+H]^+$ 317.1397; Found 317.1394.

4-Ethoxy-6-(4-methoxyphenyl)-1-phenyl-1*H*-pyrazolo[3,4-*d*]pyrimidine (5b): White solid; m.p. 152-154 °C; IR (KBr, *v*, cm^{-1}): 3102, 2990, 1591; ^{1}H NMR (400 MHz, DMSO-*d6*) (*δ*, ppm): 8.46-8.43 (m, 3H), 8.31-8.29 (m, 2H), 7.65-7.61 (m, 2H), 7.43-7.39 (m, 1H), 7.12-7.10 (m, 2H), 4.75 (q, *J* = 8.0 Hz, 2H), 3.86 (s, 3H), 1.49 (t, *J* = 8.0 Hz, 3H); ^{13}C NMR (100 MHz, DMSO-d_6) (*δ*, ppm): 163.5, 162.4, 161.9, 155.9, 139.2, 133.7, 130.6, 129.8, 129.8, 126.9, 121.1, 114.5, 102.3, 63.4, 55.8, 14.8; HRMS (ESI): calcd. For $C_{20}H_{18}N_4O_2H$ $[M+H]^+$ 347.1503; Found 347.1500.

4-Ethoxy-1-phenyl-6-(*p*-tolyl)-1*H*-pyrazolo[3,4-*d*]pyrimidine (5c): White solid; m.p. 118-120 °C; IR (KBr, *v*, cm^{-1}): 3064, 2976, 1595; ^{1}H NMR (400 MHz, DMSO-d_6) (*δ*, ppm): 8.37 (s, 1H), 8.32-8.25 (m, 4H), 7.63-7.59 (m, 2H), 7.41-7.37 (m, 1H), 7.31-7.29 (m, 2H), 4.69 (q, *J* = 8.0 Hz, 2H), 2.37 (s, 3H), 1.47 (t, *J* = 8.0 Hz, 3H); ^{13}C NMR (100 MHz, DMSO-d_6) (*δ*, ppm): 163.5, 162.0, 155.8, 141.6, 139.2, 134.6, 133.6, 129.8, 126.9, 121.2, 102.6, 63.4, 21.6, 14.8; HRMS (ESI): calcd. For $C_{20}H_{18}N_4OH$ $[M+H]^+$ 331.1553; Found 331.1550.

4-Ethoxy-1-phenyl-6-(3,4,5-trimethoxyphenyl)-1*H*-pyrazolo[3,4-*d*]pyrimidine (5d): White solid; m.p. 176-178 °C; IR (KBr, *v*, cm^{-1}): 3101, 2940, 1587; ^{1}H NMR (400 MHz, $CDCl_3$) (*δ*, ppm): 8.37 (d, *J* = 8.0 Hz, 2H), 8.20 (s, 1H), 7.88 (s, 2H), 7.58-7.54 (m, 2H), 7.38-7.35 (m, 1H), 4.79 (q, *J* = 8.0 Hz, 2H), 4.02 (s, 6H), 3.97 (s, 3H), 1.59 (t, *J* = 8.0 Hz, 3H); ^{13}C NMR (100 MHz, $CDCl_3$) (*δ*, ppm): 163.4, 161.7, 155.8, 153.1, 140.9, 139.3, 133.0, 132.8, 129.0, 126.3, 121.2, 106.1, 102.6, 62.9, 61.0, 56.1, 14.5; HRMS (ESI): calcd. For $C_{22}H_{22}N_4O_4H$ $[M+H]^+$ 407.1714; Found 407.1714.

6-(5-Bromo-2-methoxyphenyl)-4-ethoxy-1-phenyl-1*H*-pyrazolo[3,4-*d*]pyrimidine (5e): Yellow solid; m.p. 150-152 °C; IR (KBr, *v*, cm^{-1}): 3070 , 2982, 1589; ^{1}H NMR (400 MHz, DMSO-d_6) (*δ*, ppm): 8.47 (s, 1H), 8.28 (d, *J* = 8.0 Hz, 2H), 7.96 (s, 1H), 7.65 (d, *J* = 8.0

Hz, 1H), 7.60-7.56 (m, 2H), 7.39-7.36 (m, 1H), 7.18 (d, *J* = 8.0 Hz, 1H), 4.66 (q, *J* = 8.0 Hz, 2H), 3.87 (s, 3H), 1.46 (t, *J* = 8.0 Hz, 3H); ^{13}C NMR (100 MHz, DMSO-d_6) (δ, ppm): 163.2, 161.1, 157.8, 155.3, 139.1, 134.2, 133.9, 133.6, 129.8, 127.1, 121.2, 115.7, 112.1, 102.4, 63.7, 56.8, 14.7; HRMS (ESI): calcd. For $C_{20}H_{17}BrN_4O_2H$ $[M+H]^+$ 425.0608; Found 425.0601.

4-Ethoxy-6-(3-nitrophenyl)-1-phenyl-1*H*-pyrazolo[3,4-*d*]pyrimidine (5f): Pale yellow solid; m.p. 158-160 ^{o}C; IR (KBr, *v*, cm^{-1}): 3076, 2980, 1594; ^{1}H NMR (400 MHz, DMSO-d_6) (δ, ppm): 9.08-9.07 (m, 1H), 8.81-8.79 (m, 1H), 8.47 (s, 1H), 8.38-8.35 (m, 1H), 8.23-8.21 (m, 2H), 7.84-7.80 (m, 1H), 7.65-7.61 (m, 2H), 7.45-7.41 (m, 1H), 4.74 (q, *J* = 8.0 Hz, 2H), 1.51 (t, *J* = 8.0 Hz, 3H); ^{13}C NMR (100 MHz, DMSO-d_6) (δ, ppm): 163.8, 159.5, 155.4, 148.7, 138.9, 134.7, 133.8, 130.9, 129.8, 127.3, 126.1, 122.9, 121.4, 103.2, 63.9, 14.7; HRMS (ESI): calcd. For $C_{19}H_{15}N_5O_3H$ $[M+H]^+$ 362.1248; Found 362.1247.

4-Ethoxy-6-(2-nitrophenyl)-1-phenyl-1*H*-pyrazolo[3,4-*d*]pyrimidine (5g): Brown solid; m.p. 124-126 ^{o}C; IR (KBr, *v*, cm^{-1}): 3069, 2990, 1593; ^{1}H NMR (400 MHz, DMSO-d_6) (δ, ppm): 8.57 (s, 1H), 8.31-8.29 (m, 1H), 8.18-8.15 (m, 2H), 7.98-7.96 (m, 1H), 7.88-7.84 (m, 1H), 7.81-7.76 (m, 1H), 7.62-7.59 (m, 2H), 7.44-7.41 (m, 1H), 4.64 (q, *J* = 8.0 Hz, 2H), 1.46 (t, *J* = 8.0 Hz, 3H); ^{13}C NMR (100 MHz, DMSO-d_6) (δ, ppm): 163.6, 159.6, 155.0, 150.6, 138.7, 133.9, 132.7, 132.1, 132.0, 131.3, 129.8, 127.4, 124.3, 121.4, 102.8, 64.0, 14.7; HRMS (ESI): calcd. For $C_{19}H_{15}N_5O_3H$ $[M+H]^+$ 362.1248; Found 362.1247.

1-(4-Chlorophenyl)-4-ethoxy-6-phenyl-1*H*-pyrazolo[3,4-*d*]pyrimidine (5h): Yellow solid; m.p. 160-162 ^{o}C; IR (KBr, *v*, cm^{-1}): 3108, 2984, 1591; ^{1}H NMR (400 MHz, DMSO-d_6) (δ, ppm): 8.50-8.47 (m, 3H), 8.34-8.32 (m, 2H), 7.69-7.67 (m, 2H), 7.57-7.56 (m, 3H), 4.76 (q, *J* = 8.0 Hz, 2H), 1.50 (t, *J* = 8.0 Hz, 3H); ^{13}C NMR (100 MHz, DMSO-d_6) (δ, ppm):

163.6, 162.0, 155.9, 138.0, 137.2, 134.0, 131.8, 131.1, 129.8, 129.1, 128.9, 122.6, 102.8, 63.6, 14.8; HRMS (ESI): calcd. For $C_{19}H_{15}ClN_4OH$ $[M+H]^+$ 351.1007; Found 351.1006.

1-(3,5-Dimethylphenyl)-4-ethoxy-6-phenyl-1*H*-pyrazolo[3,4-*d*]pyrimidine (5i): Yellow solid; m.p. 156-158 °C; IR (KBr, *v*, cm^{-1}): 3107, 2980, 1596; ^1H NMR (400 MHz, DMSO-d_6) (*δ*, ppm): 8.49-8.46 (m, 2H), 8.43 (s, 1H), 7.91 (s, 2H), 7.58-7.56 (m, 3H), 7.05 (s, 1H), 4.77 (q, *J* = 8.0 Hz, 2H), 2.42 (s, 6H), 1.50 (t, *J* = 8.0 Hz, 3H); ^{13}C NMR (100 MHz, DMSO-d_6) (*δ*, ppm): 163.6, 161.8, 139.0, 137.4, 136.3, 133.4, 131.7, 129.3, 129.2, 128.8, 128.6, 119.9, 119.1, 102.7, 63.5, 21.7, 21.6, 14.8; HRMS (ESI): calcd. For $C_{21}H_{20}N_4OH$ $[M+H]^+$ 345.1710; Found 345.1707.

4-Ethoxy-1-methyl-6-phenyl-1*H*-pyrazolo[3,4-*d*]pyrimidine (5j): Yellow solid; m.p. 214-216 °C; IR (KBr, *v*, cm^{-1}): 3092, 2984, 1617; ^1H NMR (400 MHz, DMSO-d_6) (*δ*, ppm): 8.60 (s, 1H), 8.47-8.44 (m, 2H), 7.53-7.51 (m, 3H), 4.72 (q, *J* = 8.0 Hz, 2H), 4.15 (s, 3H), 1.47 (t, *J* = 8.0 Hz, 3H); ^{13}C NMR (100 MHz, DMSO-d_6) (*δ*, ppm): 164.6, 162.8, 160.4, 138.4, 130.9, 128.9, 128.5, 125.8, 101.7, 62.9, 41.0, 14.8; HRMS (ESI): calcd. For $C_{14}H_{14}N_4OH$ $[M+H]^+$ 255.1240; Found 255.1240.

4-Ethoxy-1,3-dimethyl-6-phenyl-1*H*-pyrazolo[3,4-*d*]pyrimidine (5k): Yellow solid; m.p. 136-138 °C; IR (KBr, *v*, cm^{-1}): 2984, 1598; ^1H NMR (400 MHz, DMSO-d_6) (*δ*, ppm): 8.50-8.47 (m, 2H), 7.55-7.54 (m, 3H), 4.71 (q, *J* = 8.0 Hz, 2H), 3.97 (s, 3H), 2.09 (s, 3H), 1.47 (t, *J* = 8.0 Hz, 3H); ^{13}C NMR (100 MHz, DMSO-d_6) (*δ*, ppm): 164.0, 161.0, 156.5, 140.8, 137.6, 131.3, 129.0, 128.7, 100.0, 62.9, 33.8, 14.8, 14.1; HRMS (ESI): calcd. For $C_{15}H_{16}N_4OH$ $[M+H]^+$ 269.1397; Found 269.1393.

4-Methoxy-6-(4-methoxyphenyl)-1-phenyl-1*H*-pyrazolo[3,4-*d*]pyrimidine (5l): White solid; m.p. 142-144 °C; IR (KBr, *v*, cm^{-1}): 3097, 2956, 1595; ^1H NMR (400 MHz, DMSO-d_6)

(δ, ppm): 8.47-8.45 (m, 3H), 8.31-8.29 (m, 2H), 7.65-7.61 (m, 2H), 7.43-7.39 (m, 1H), 7.12-7.10 (m, 2H), 4.24 (s, 3H), 3.86 (s, 3H); ^{13}C NMR (100 MHz, DMSO-d_6) (δ, ppm): 163.8, 162.4, 161.9, 155.9, 139.2, 133.6, 130.6, 129.8, 127.0, 121.2, 114.5, 102.3, 55.9, 54.5; HRMS (ESI): calcd. For $C_{19}H_{16}N_4O_2H$ [M+H]$^+$ 333.1346; Found 333.1342.

4-Methoxy-6-(3-nitrophenyl)-1-phenyl-1*H*-pyrazolo[3,4-*d*]pyrimidine (5m): White solid; m.p. 175-177 ^{o}C; IR (KBr, *v*, cm^{-1}): 3113, 2935, 1595; ^{1}H NMR (400 MHz, DMSO-d_6) (δ, ppm): 8.92 (m, 1H), 8.67-8.65 (m, 1H), 8.35 (s, 1H), 8.29-8.26 (m, 1H), 8.14-8.12 (m, 2H), 7.74-7.70 (m, 1H), 7.58-7.54 (m, 2H), 7.41-7.37 (m, 1H), 4.15 (s, 3H); ^{13}C NMR (100 MHz, DMSO-d_6) (δ, ppm): 164.2, 159.7, 155.4, 148.7, 138.9, 134.9, 133.8, 131.0, 129.9, 127.4, 126.2, 123.0, 121.6, 103.2, 55.0; HRMS (ESI): calcd. For $C_{18}H_{13}N_5O3H$ [M+H]$^+$ 348.1091; Found 348.1091.

4-Butoxy-1,6-diphenyl-1*H*-pyrazolo[3,4-*d*]pyrimidine (5n): White solid; m.p. 99-101 ^{o}C; IR (KBr, *v*, cm^{-1}): 3065, 2956, 1594; ^{1}H NMR (400 MHz, DMSO-d_6) (δ, ppm): 8.49-8.47 (m, 2H), 8.45 (s, 1H), 8.30-8.28 (m, 2H), 7.65-7.61 (m, 2H), 7.57-7.55 (m, 3H), 7.43-7.39 (m, 1H), 4.70 (t, J = 8.0 Hz, 2H), 1.89-1.82 (m, 2H), 1.56-1.47 (m, 2H), 0.99 (t, J = 8.0 Hz, 3H); ^{13}C NMR (100 MHz, DMSO-d_6) (δ, ppm): 163.7, 161.9, 155.8, 139.1, 137.3, 133.6, 131.7, 129.8, 128.8, 127.0, 121.1, 102.7, 67.1, 30.8, 19.2, 14.2; HRMS (ESI): calcd. For $C_{21}H_{20}N_4OH$ [M+H]$^+$ 345.1710; Found 345.1708.

1,6-Diphenyl-4-propoxy-1*H*-pyrazolo[3,4-*d*]pyrimidine (5o): White solid; m.p. 128-129 ^{o}C; IR (KBr, *v*, cm^{-1}): 3066, 2966, 1293; ^{1}H NMR (400 MHz, DMSO-d_6) (δ, ppm): 8.49-8.47 (m, 2H), 8.46 (s, 1H), 8.31-8.28 (m, 2H), 7.65-7.61 (m, 2H), 7.57-7.55 (m, 3H), 7.43-7.39 (m, 1H), 4.66 (t, J = 8.0 Hz, 2H), 1.95-1.86 (m, 2H), 1.07 (t, J = 8.0 Hz, 3H); ^{13}C NMR (100 MHz, DMSO-d_6) (δ, ppm): 163.8, 161.9, 155.8, 139.1, 137.3, 133.6, 131.7, 129.8, 129.1,

128.8, 127.0, 121.2, 102.7, 68.9, 22.2, 10.8; HRMS (ESI): calcd. For $C_{20}H_{18}N_4OH$ $[M+H]^+$ 331.1553; Found 331.1551.

4-Isopropoxy-1,6-diphenyl-1*H*-pyrazolo[3,4-*d*]pyrimidine (5p): White solid; m.p. 192-194 ^oC; IR (KBr, v, cm^{-1}): 3064, 2977, 1595; ^1H NMR (400 MHz, DMSO-d_6) (δ, ppm): 8.53-8.51 (m, 2H), 8.48 (s, 1H), 8.33-8.31 (m, 2H), 7.67-7.63 (m, 2H), 7.60-7.58 (m, 3H), 7.45-7.41 (m, 1H), 5.87-5.81 (m, 1H), 1.52 (d, J = 8 Hz, 6H); ^{13}C NMR (100 MHz, DMSO-d_6) (δ, ppm): 163.6, 162.0, 155.9, 139.2, 137.5, 133.7, 131.2, 130.7, 129.9, 129.6, 129.2, 128.8, 127.1, 121.3, 100.0, 78.2, 22.2; HRMS (ESI): calcd. For $C_{20}H_{18}N_4OH$ $[M+H]^+$ 331.1553; Found 331.1549.

^{1}H and ^{13}C NMR spectra of 5

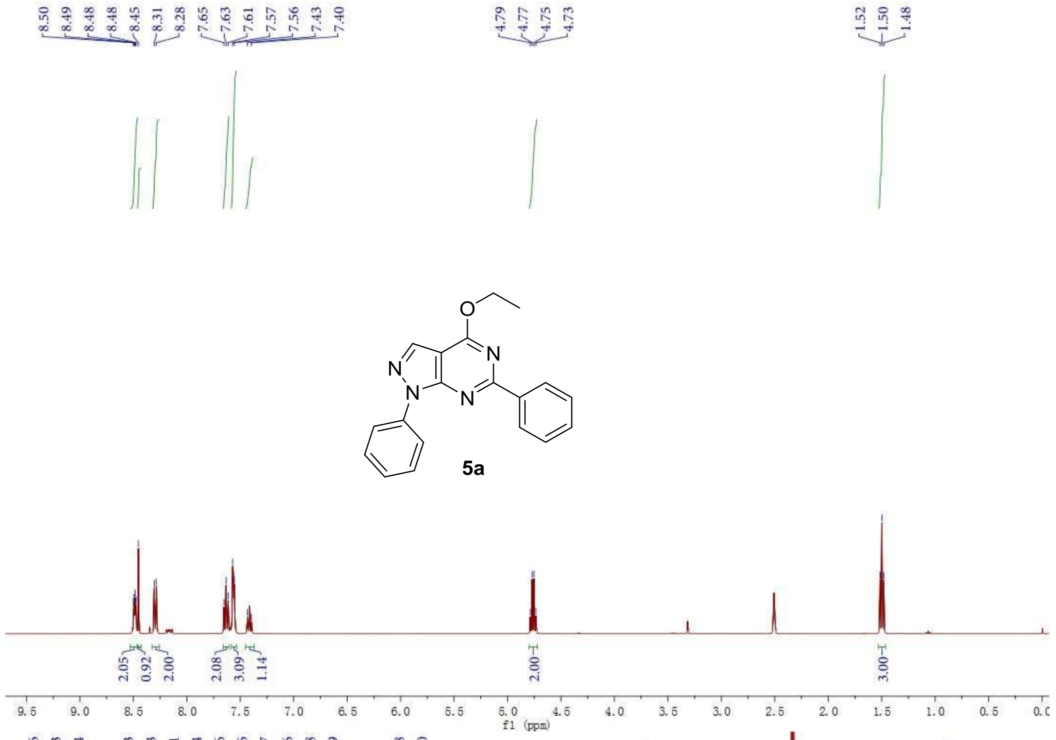

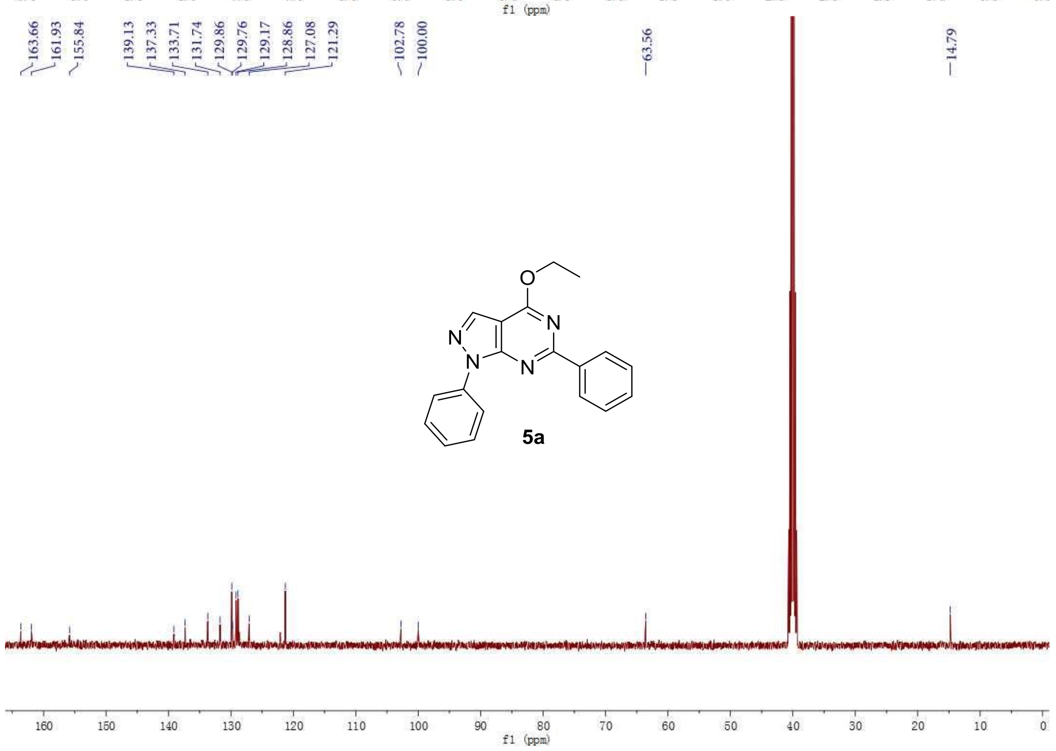

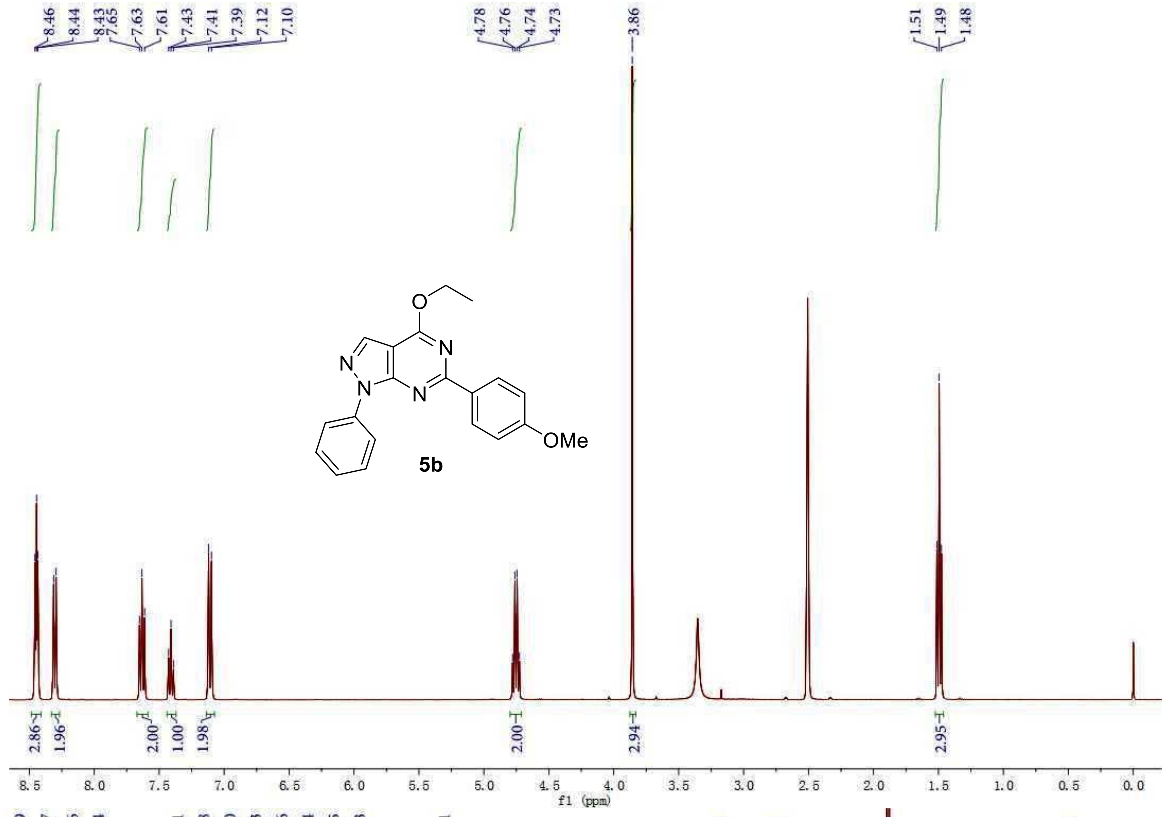
8.46 8.44 8.43 7.65 7.63 7.61 7.43 7.41 7.39 7.12 7.10
4.78 4.76 4.74 4.73
3.86
1.51 1.49 1.48
N
N
N
N
O
OMe
5b
2.86 1.96 2.00 1.00 1.98 2.00 2.94 2.95
8.5 8.0 7.5 7.0 6.5 6.0 5.5 5.0 4.5 4.0 3.5 3.0 2.5 2.0 1.5 1.0 0.5 0.0
f1 (ppm)

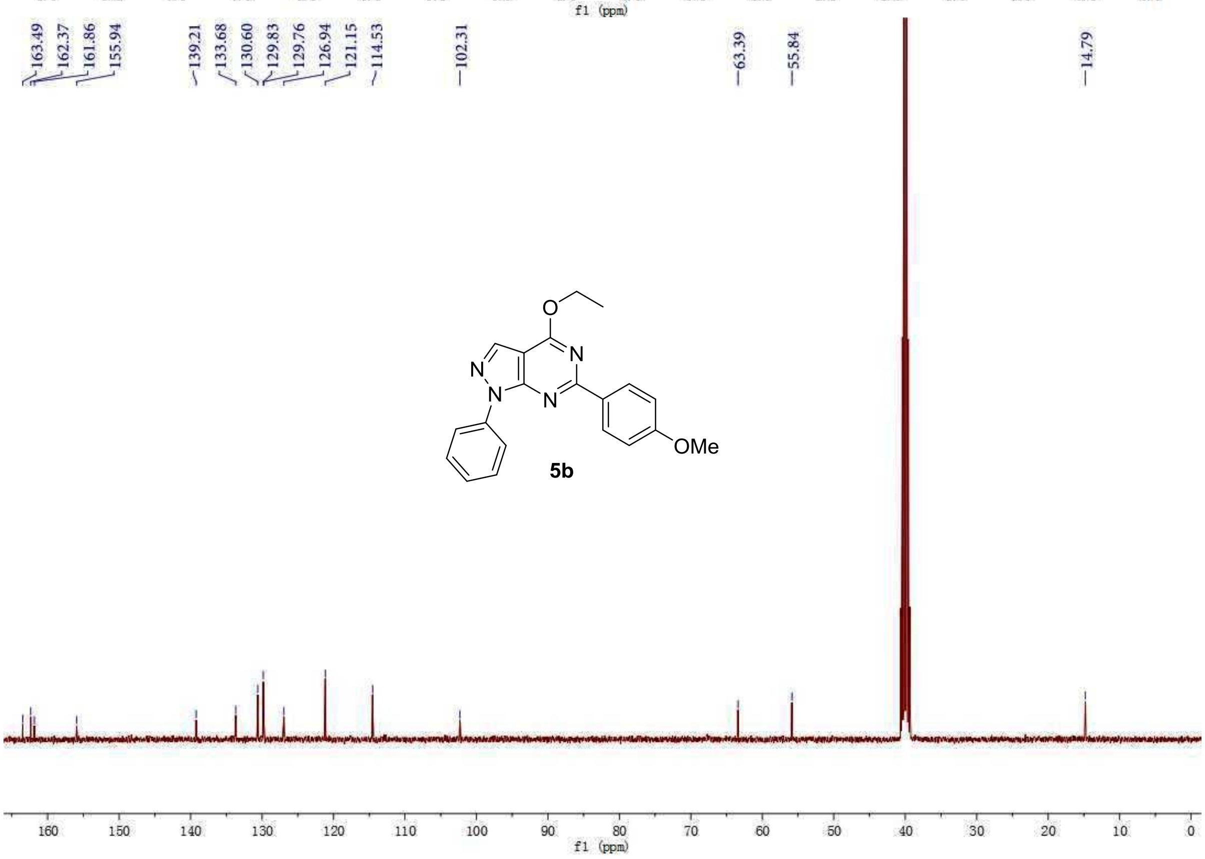
163.49 162.37 161.86 155.94
139.21 133.68 130.60 129.83 129.76 126.94 121.15 114.53
102.31
63.39
55.84
14.79
N
N
N
N
O
OMe
5b
160 150 140 130 120 110 100 90 80 70 60 50 40 30 20 10 0
f1 (ppm)

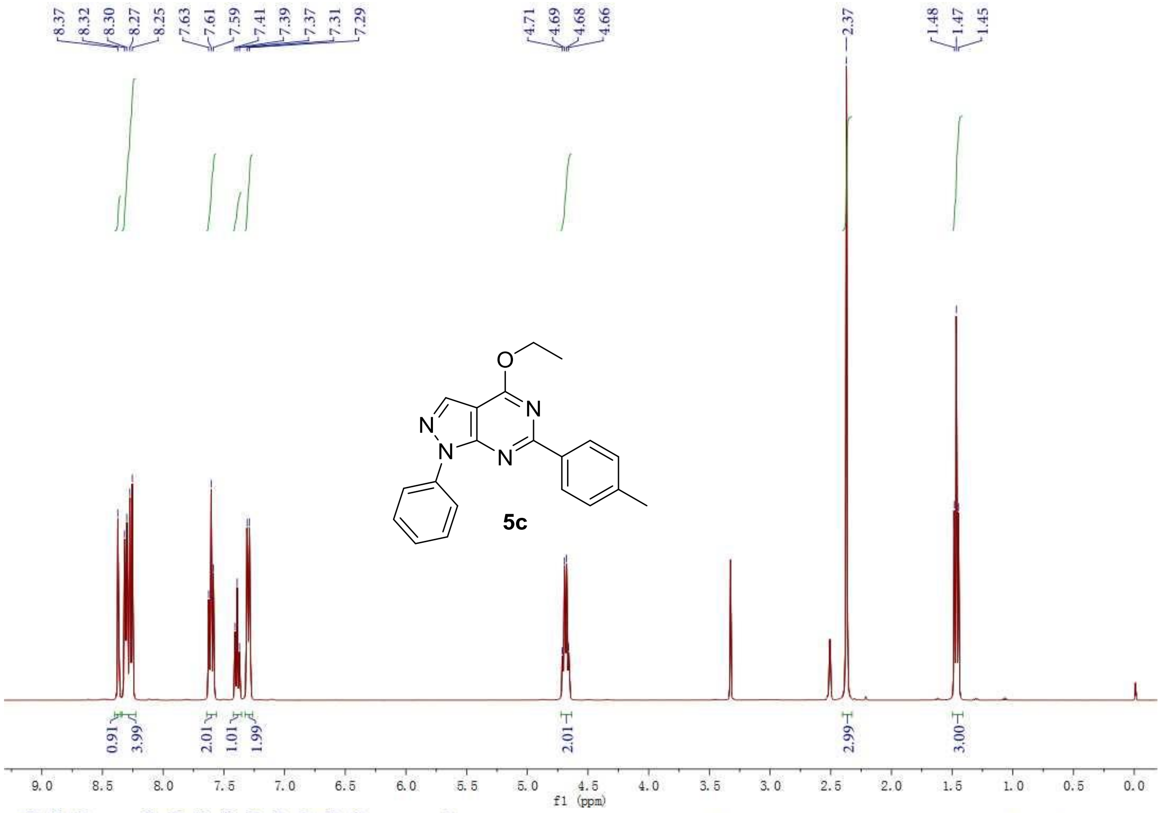
8.37
8.32
8.30
8.27
8.25
7.63
7.61
7.59
7.41
7.39
7.37
7.31
7.29
4.71
4.69
4.68
4.66
2.37
1.48
1.47
1.45
5c
0.91
3.99
2.01
1.01
1.99
2.01
2.99
3.00
f1 (ppm)

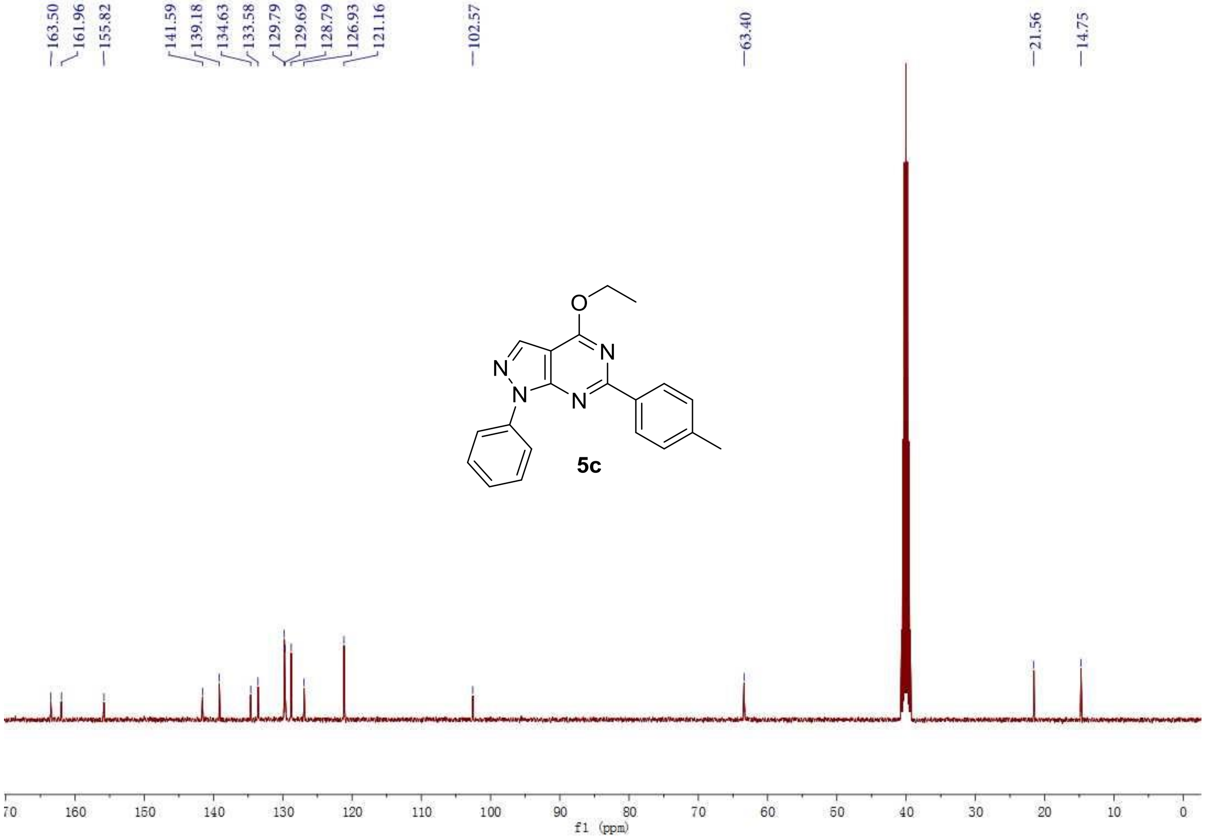
163.50
161.96
155.82
141.59
139.18
134.63
133.58
129.79
129.69
128.79
126.93
121.16
102.57
63.40
21.56
14.75
5c
f1 (ppm)

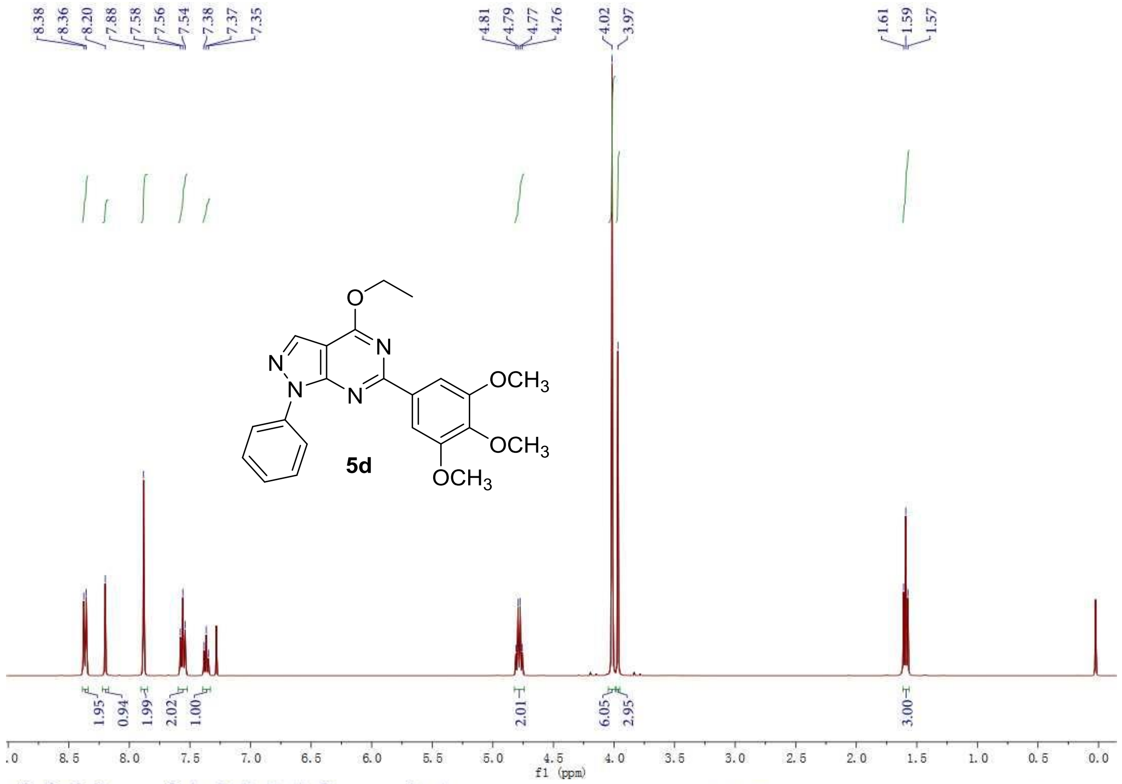
8.38
8.36
8.20
7.88
7.58
7.56
7.54
7.38
7.37
7.35
4.81
4.79
4.77
4.76
4.02
3.97
1.61
1.59
1.57
N
N
N
N
O
OCH$_3$
OCH$_3$
OCH$_3$
5d
1.95
0.94
1.99
2.02
1.00
2.01
6.05
2.95
3.00
f1 (ppm)

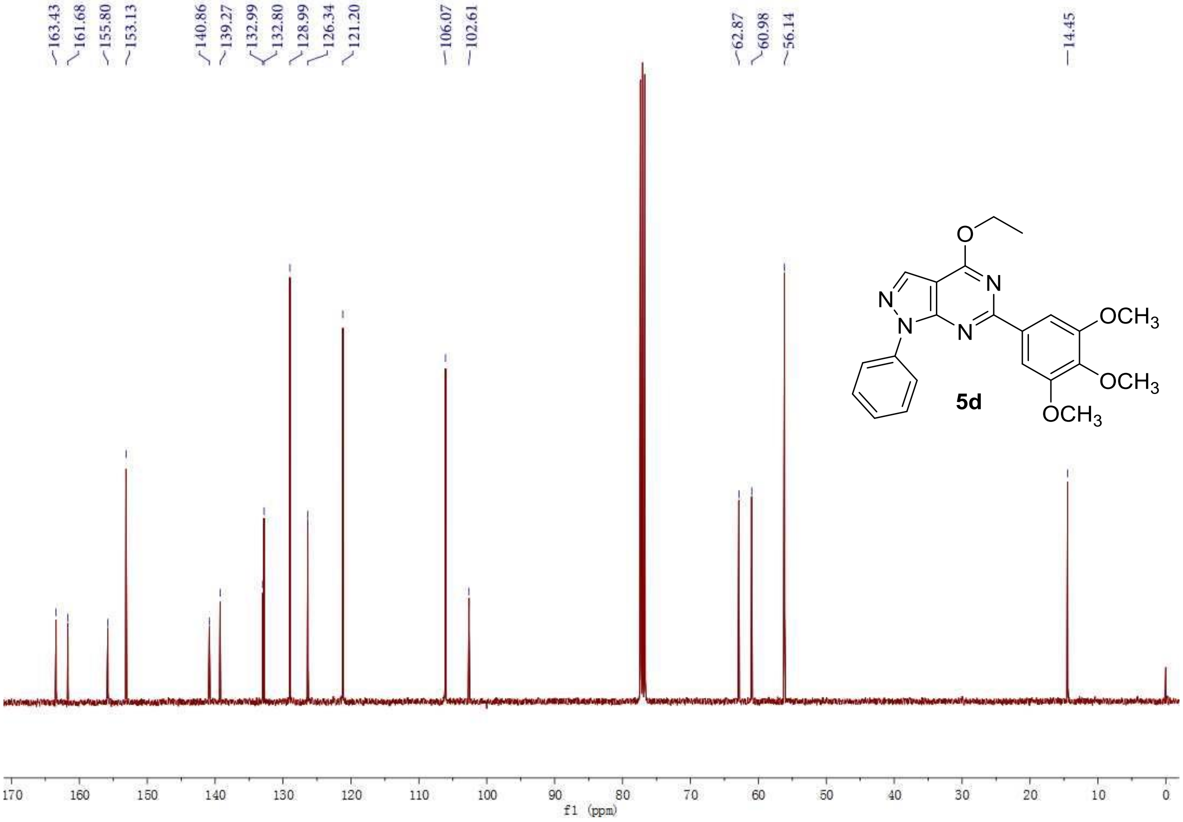
163.43
161.68
155.80
153.13
140.86
139.27
132.99
132.80
128.99
126.34
121.20
106.07
102.61
62.87
60.98
56.14
14.45
N
N
N
N
O
OCH$_3$
OCH$_3$
OCH$_3$
5d
f1 (ppm)

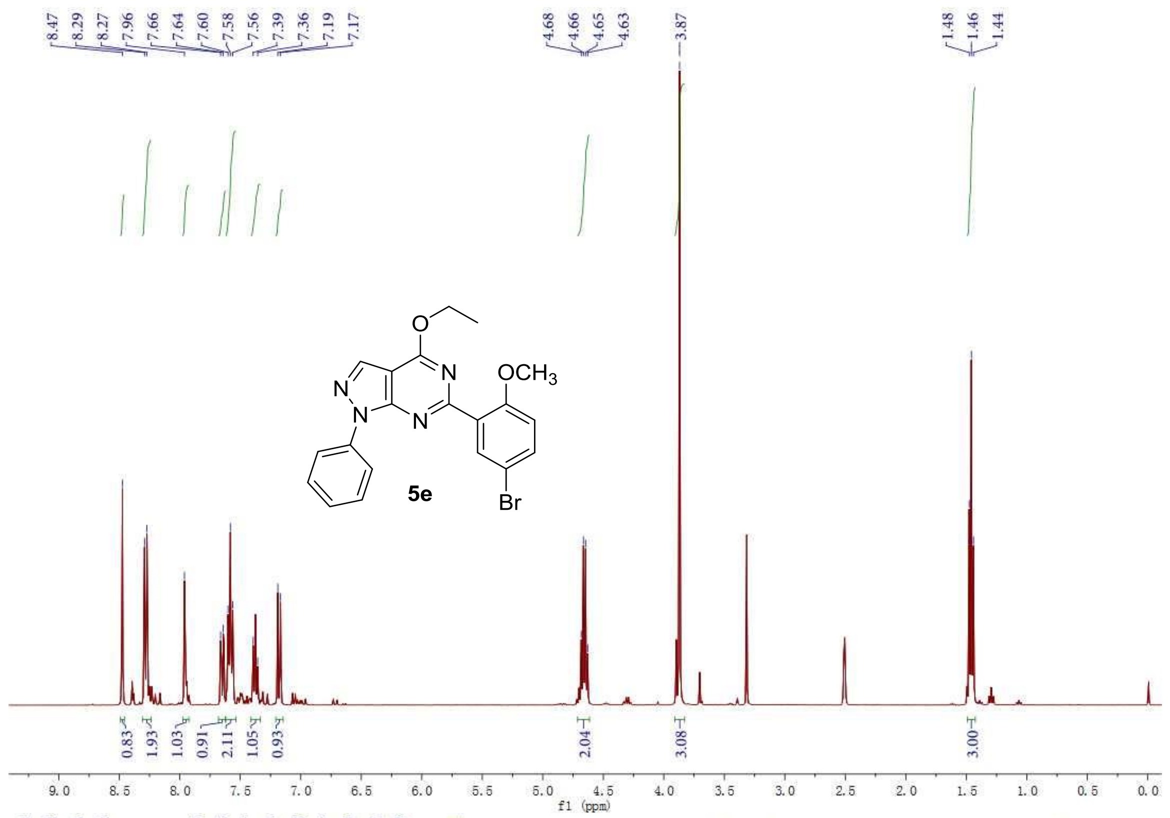

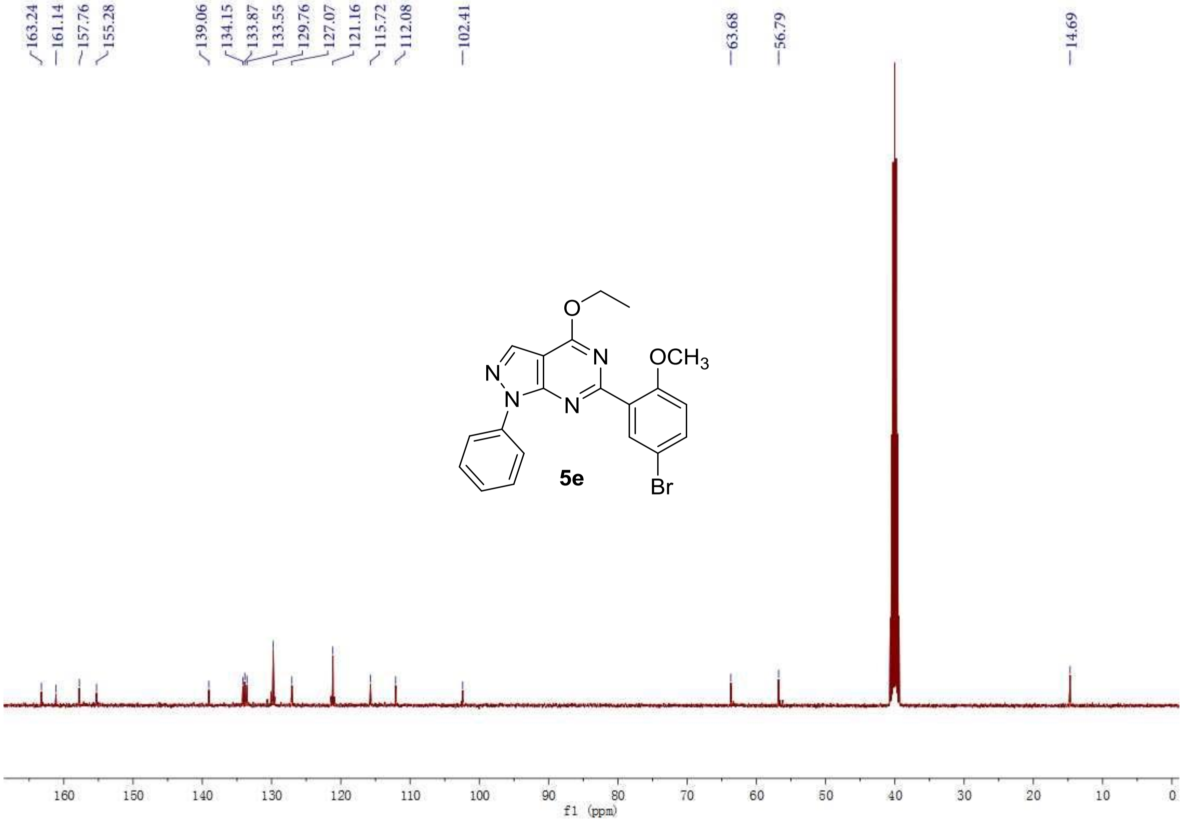

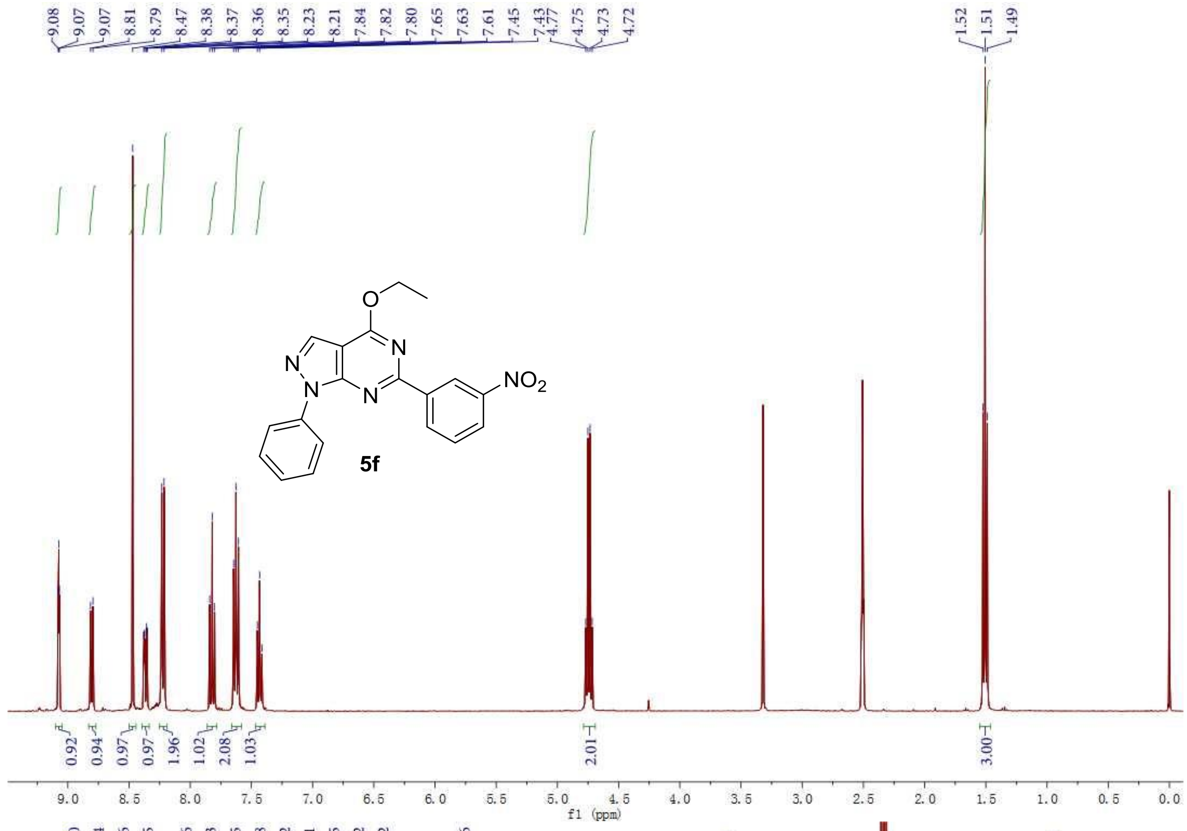
9.08 9.07 9.07 8.81 8.79 8.47 8.38 8.37 8.36 8.35 8.23 8.21 7.84 7.82 7.80 7.65 7.63 7.61 7.45 7.43 4.77 4.75 4.73 4.72
1.52 1.51 1.49
0.92 0.94 0.97 0.97 1.96 1.02 2.08 1.03 2.01 3.00
N
N
O
N
N
NO2
5f
f1 (ppm)

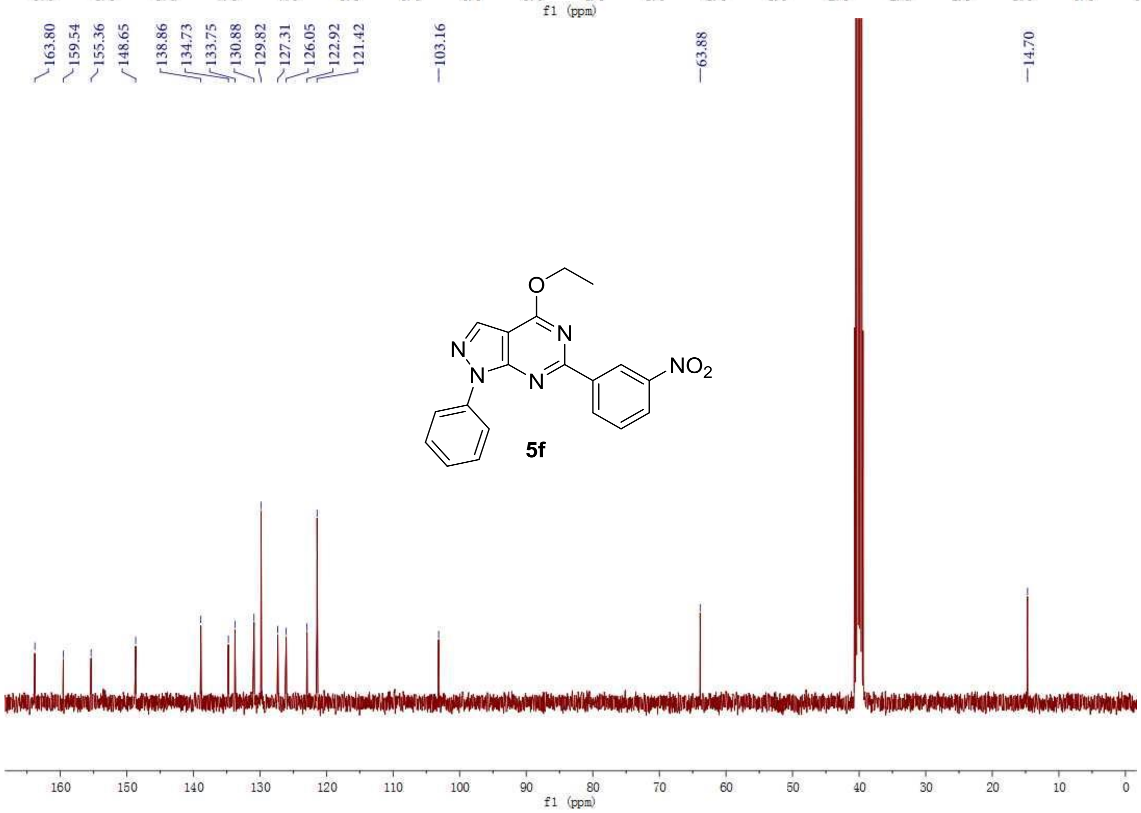
163.80 159.54 155.36 148.65 138.86 134.73 133.75 130.88 129.82 127.31 126.05 122.92 121.42
103.16
63.88
14.70
N
N
O
N
N
NO2
5f
f1 (ppm)

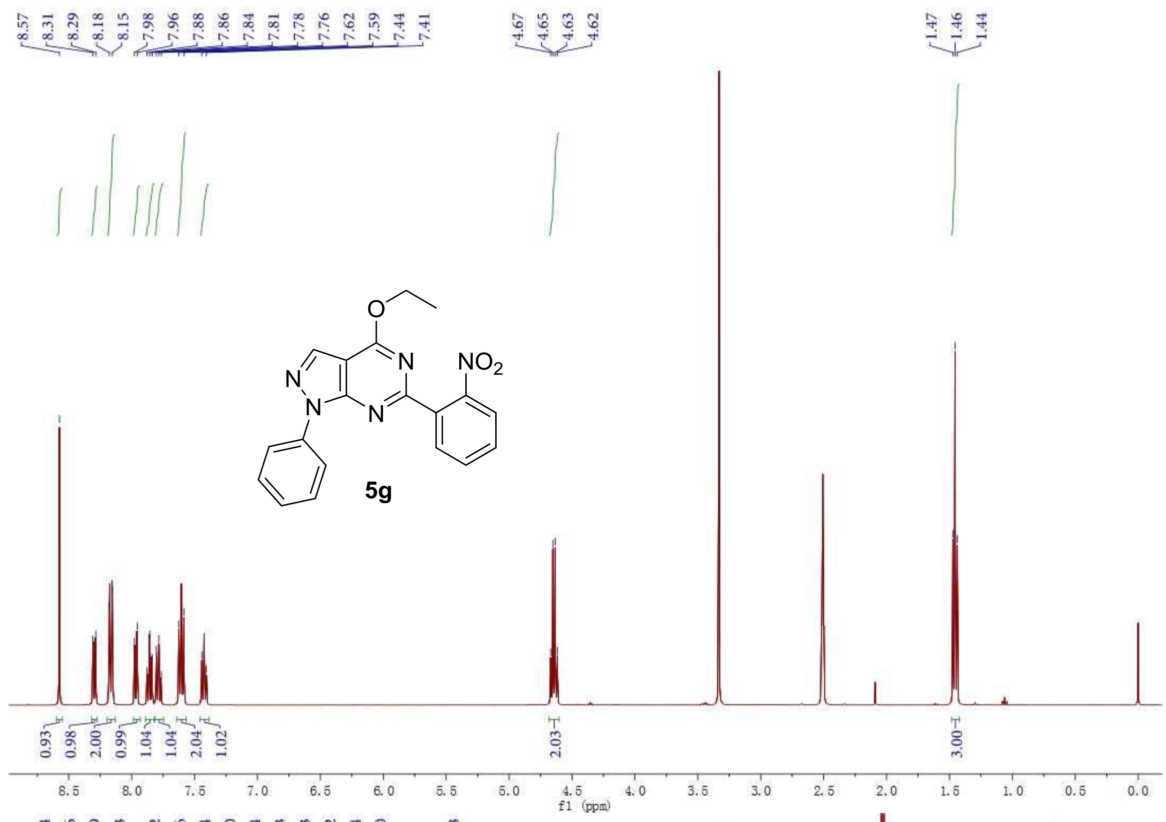

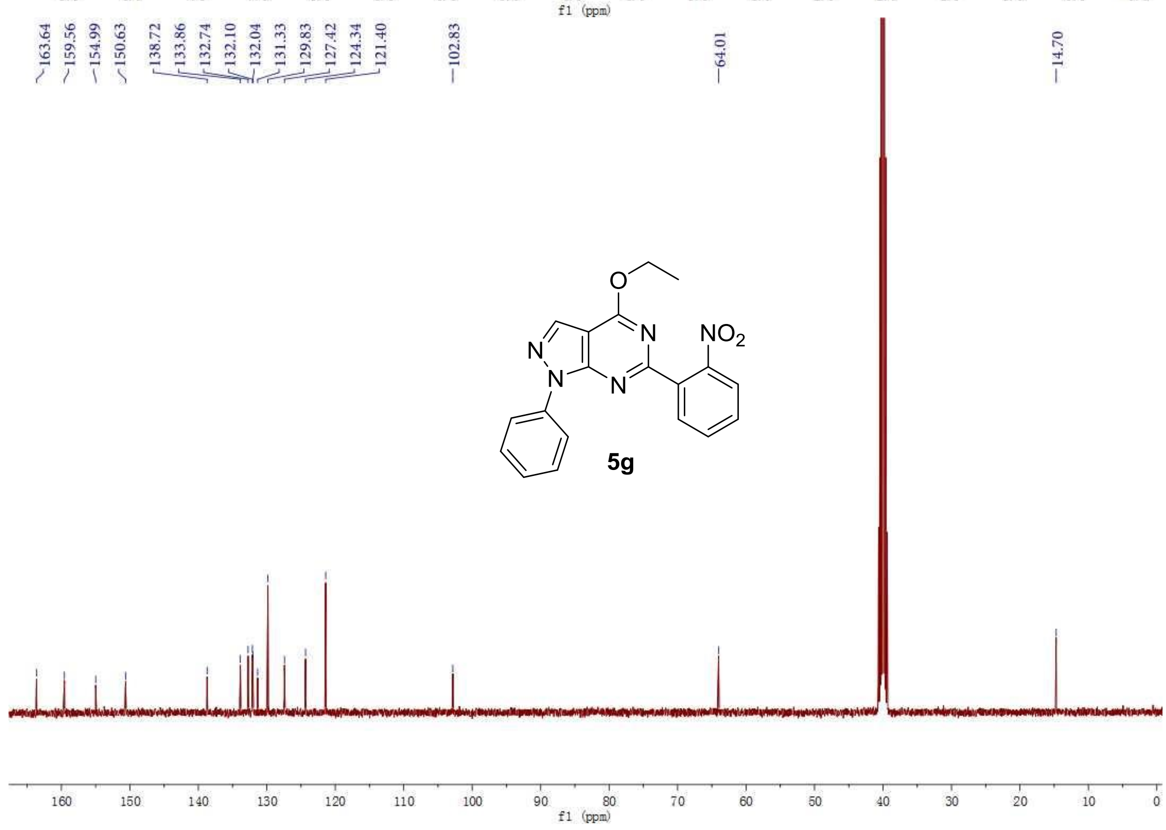

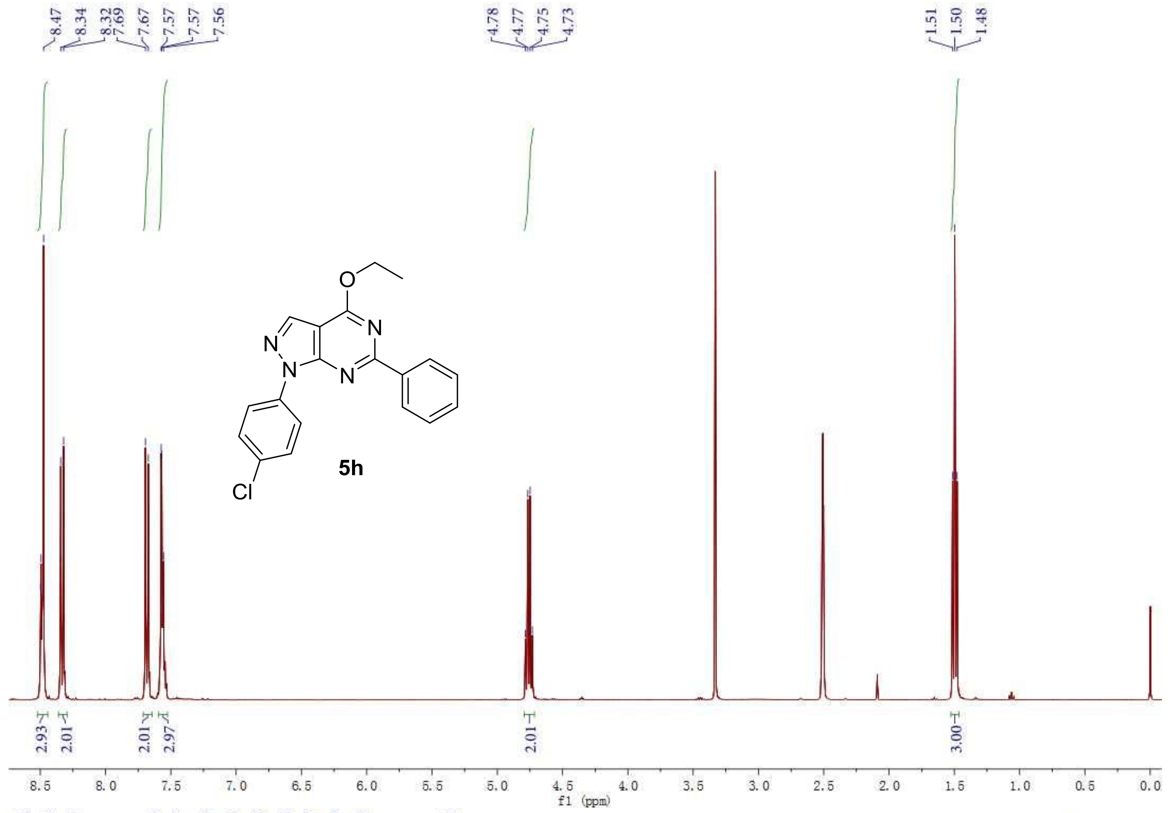
8.47
8.34
8.32
7.69
7.67
7.57
7.57
7.56
4.78
4.77
4.75
4.73
1.51
1.50
1.48
N
N
O
N
N
Cl
5h
2.93
2.01
2.01
2.97
2.01
3.00
8.5 8.0 7.5 7.0 6.5 6.0 5.5 5.0 4.5 4.0 3.5 3.0 2.5 2.0 1.5 1.0 0.5 0.0
f1 (ppm)

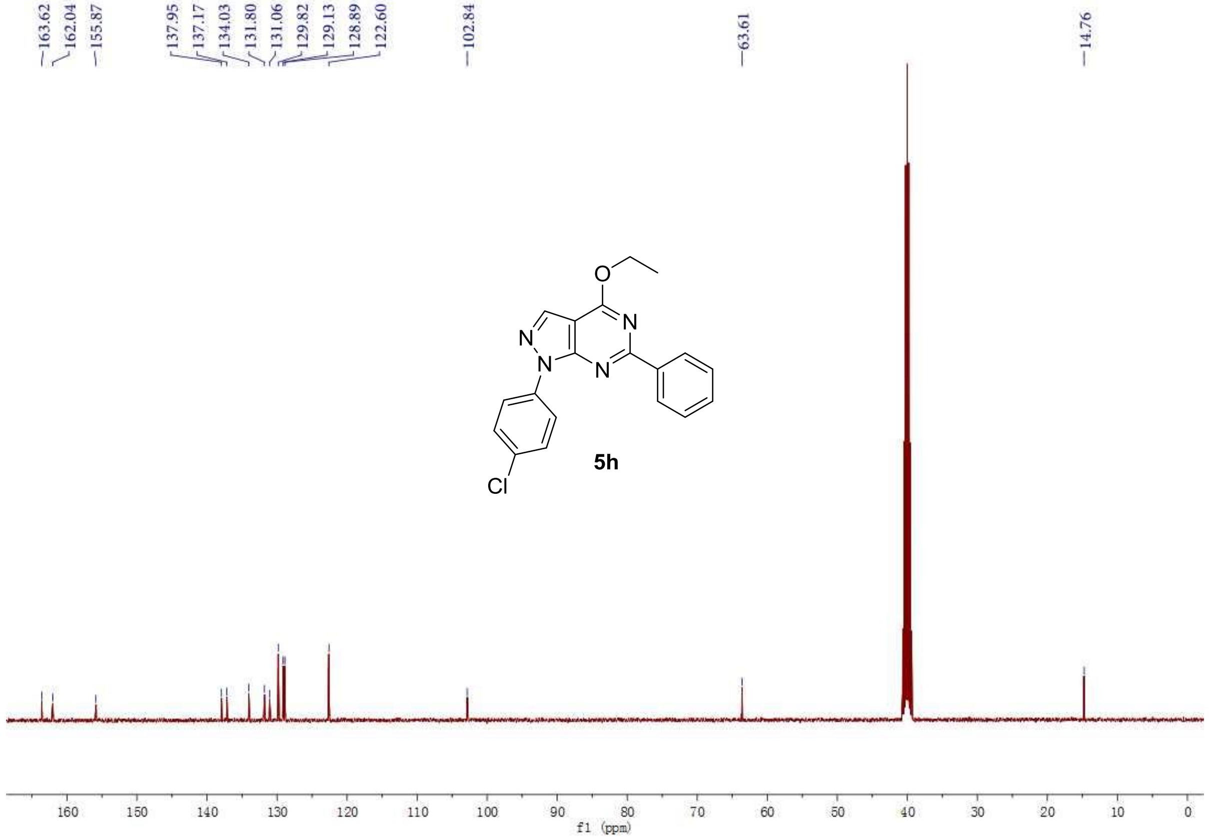
163.62
162.04
155.87
137.95
137.17
134.03
131.80
131.06
129.82
129.13
128.89
122.60
102.84
63.61
14.76
N
N
O
N
N
Cl
5h
160 150 140 130 120 110 100 90 80 70 60 50 40 30 20 10 0
f1 (ppm)

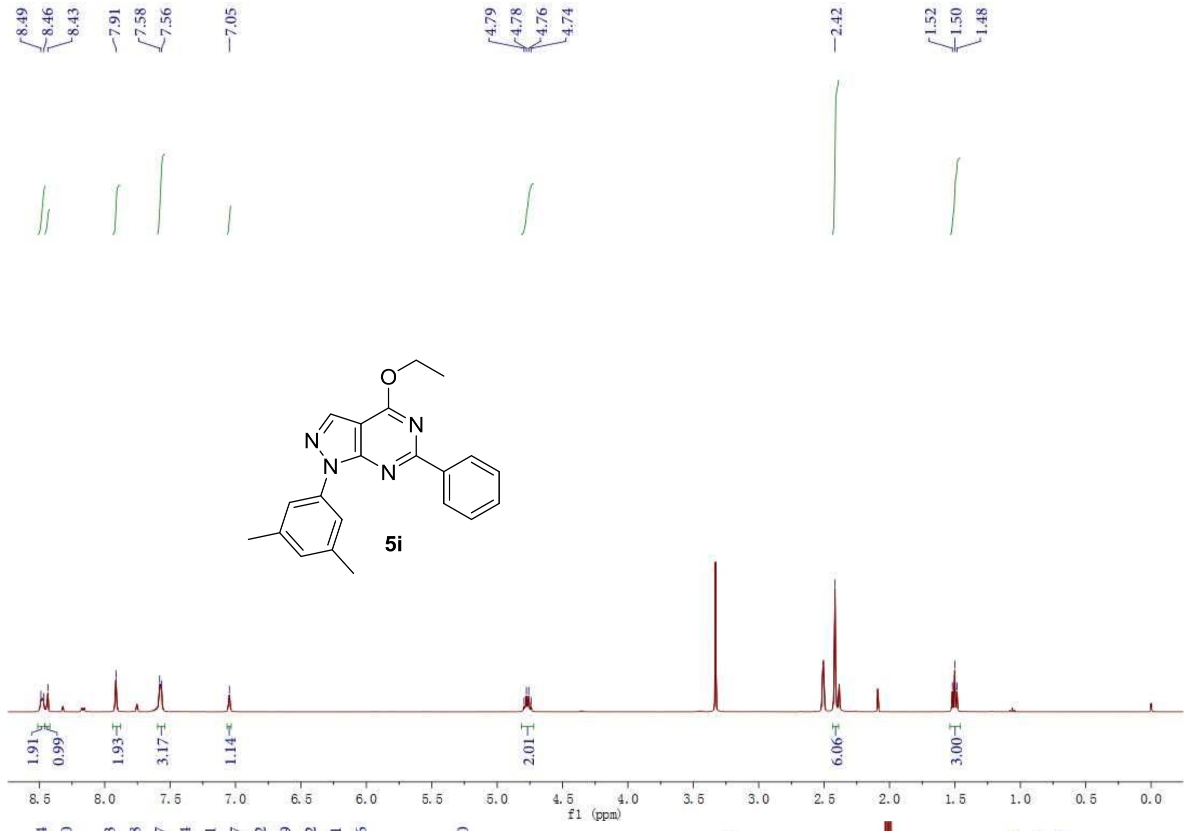
8.49
8.46
8.43
7.91
7.58
7.56
7.05
4.79
4.78
4.76
4.74
2.42
1.52
1.50
1.48
5i
1.91
0.99
1.93
3.17
1.14
2.01
6.06
3.00
f1 (ppm)

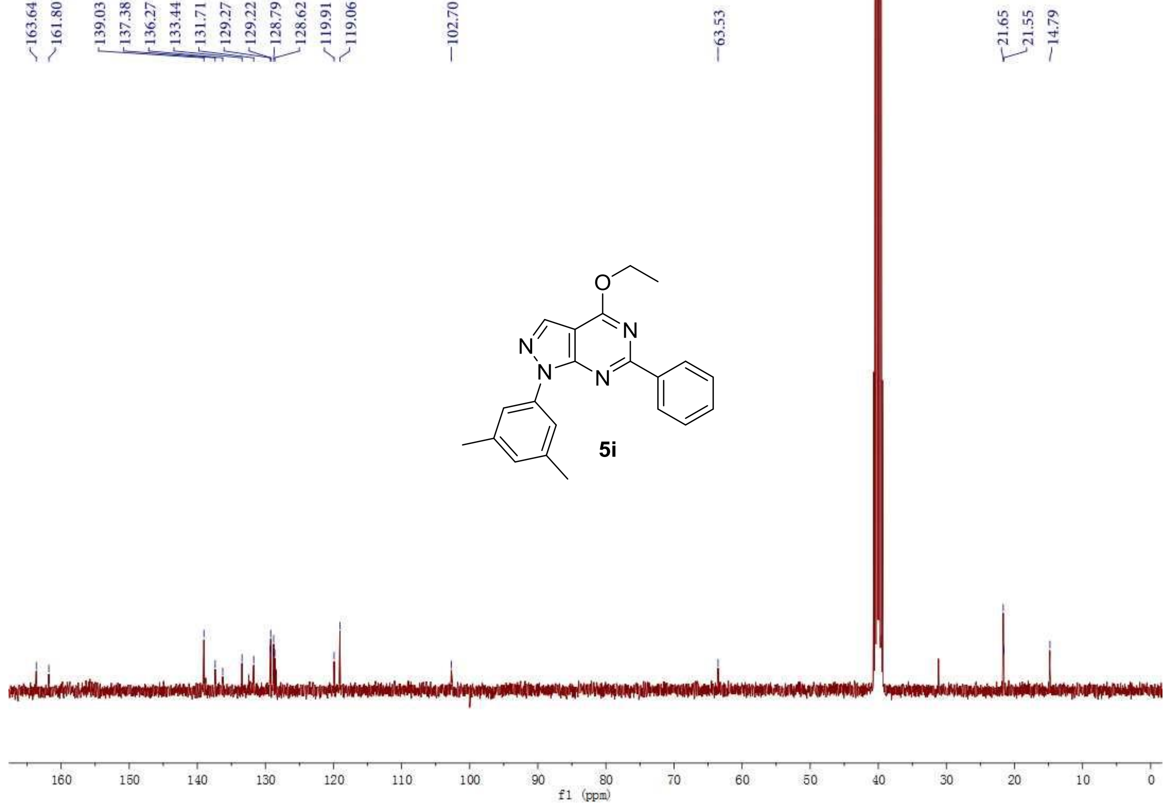
163.64
161.80
139.03
137.38
136.27
133.44
131.71
129.27
129.22
128.79
128.62
119.91
119.06
102.70
63.53
21.65
21.55
14.79
5i
f1 (ppm)

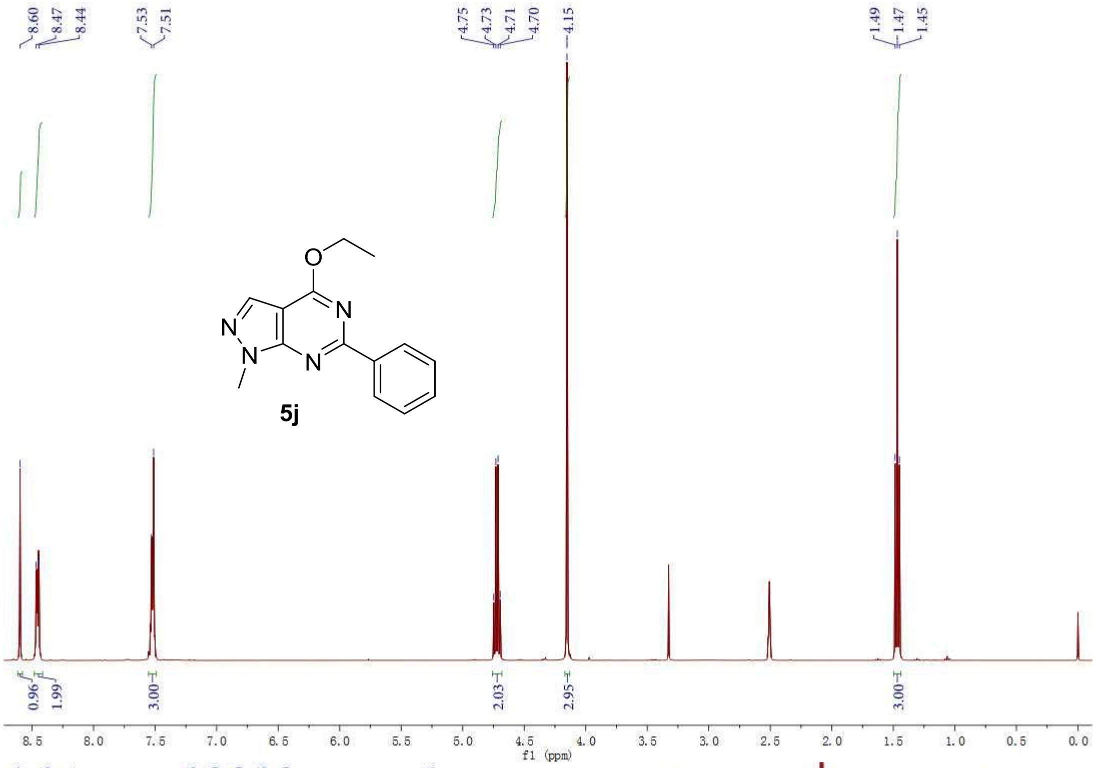
8.60
8.47
8.44
7.53
7.51
4.75
4.73
4.71
4.70
4.15
1.49
1.47
1.45
5j
0.96
1.99
3.00
2.03
2.95
3.00
f1 (ppm)

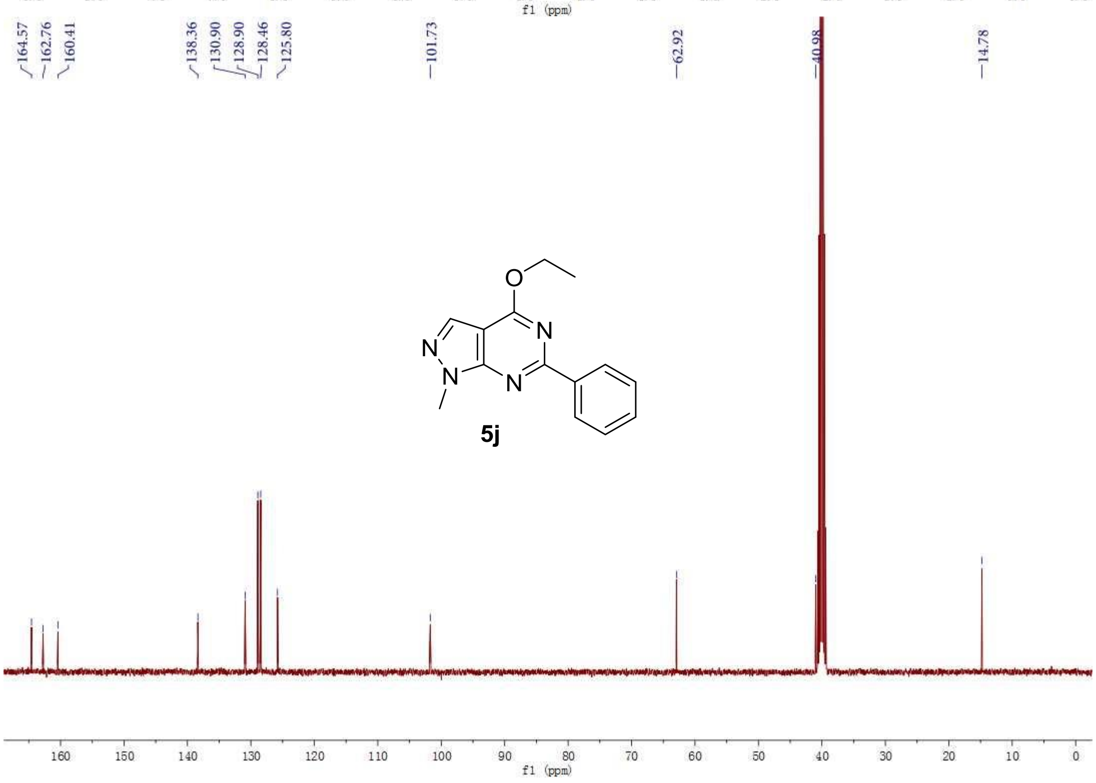
164.57
162.76
160.41
138.36
130.90
128.90
128.46
125.80
101.73
62.92
40.98
14.78
5j
f1 (ppm)

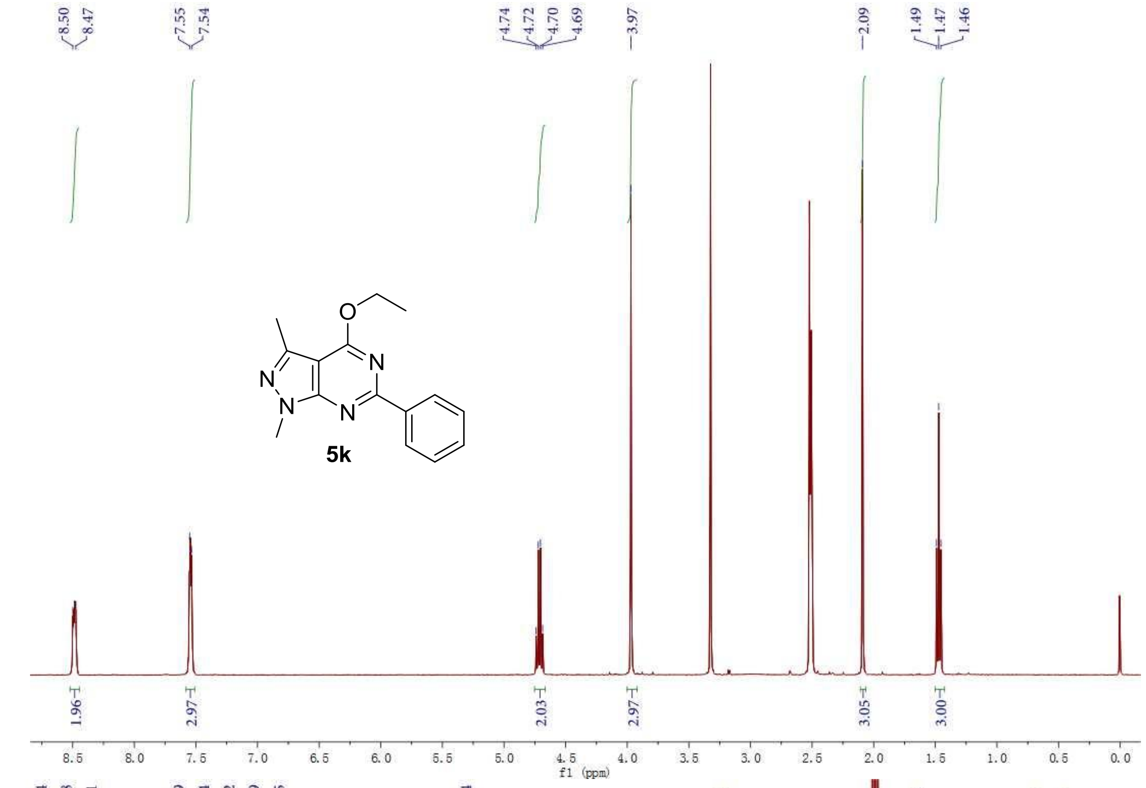
8.50
8.47
7.55
7.54
4.74
4.72
4.70
4.69
3.97
2.09
1.49
1.47
1.46
5k
1.96
2.97
2.03
2.97
3.05
3.00
f1 (ppm)

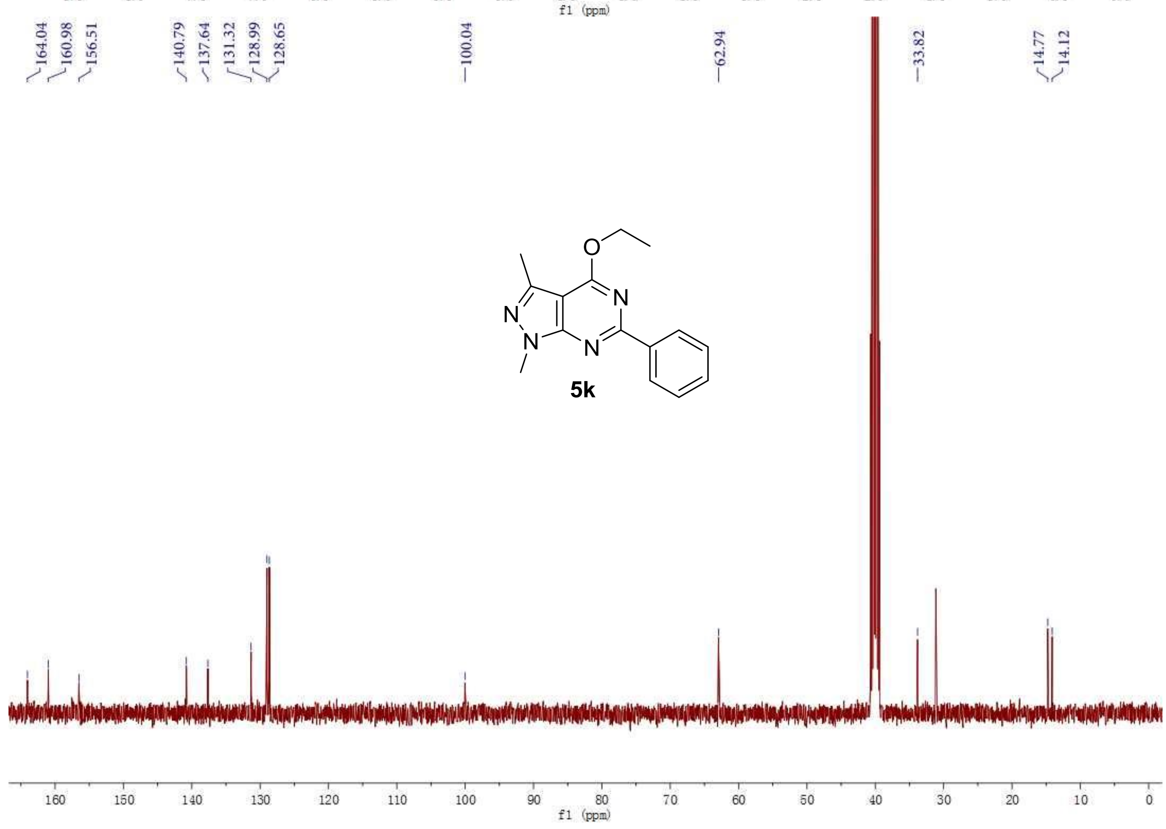
164.04
160.98
156.51
140.79
137.64
131.32
128.99
128.65
100.04
62.94
33.82
14.77
14.12
5k
f1 (ppm)

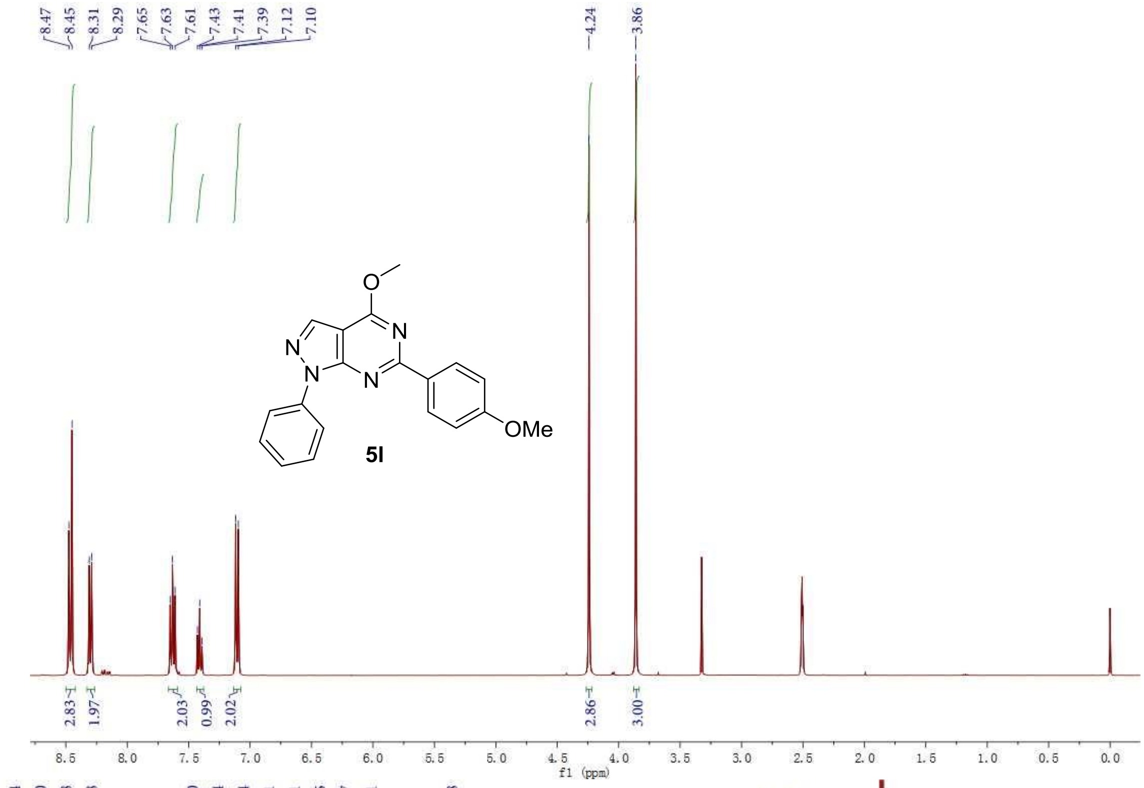
8.47
8.45
8.31
8.29
7.65
7.63
7.61
7.43
7.41
7.39
7.12
7.10
4.24
3.86
N
N
N
N
O
OMe
5l
2.83
1.97
2.03
0.99
2.02
2.86
3.00
8.5 8.0 7.5 7.0 6.5 6.0 5.5 5.0 4.5 4.0 3.5 3.0 2.5 2.0 1.5 1.0 0.5 0.0
f1 (ppm)

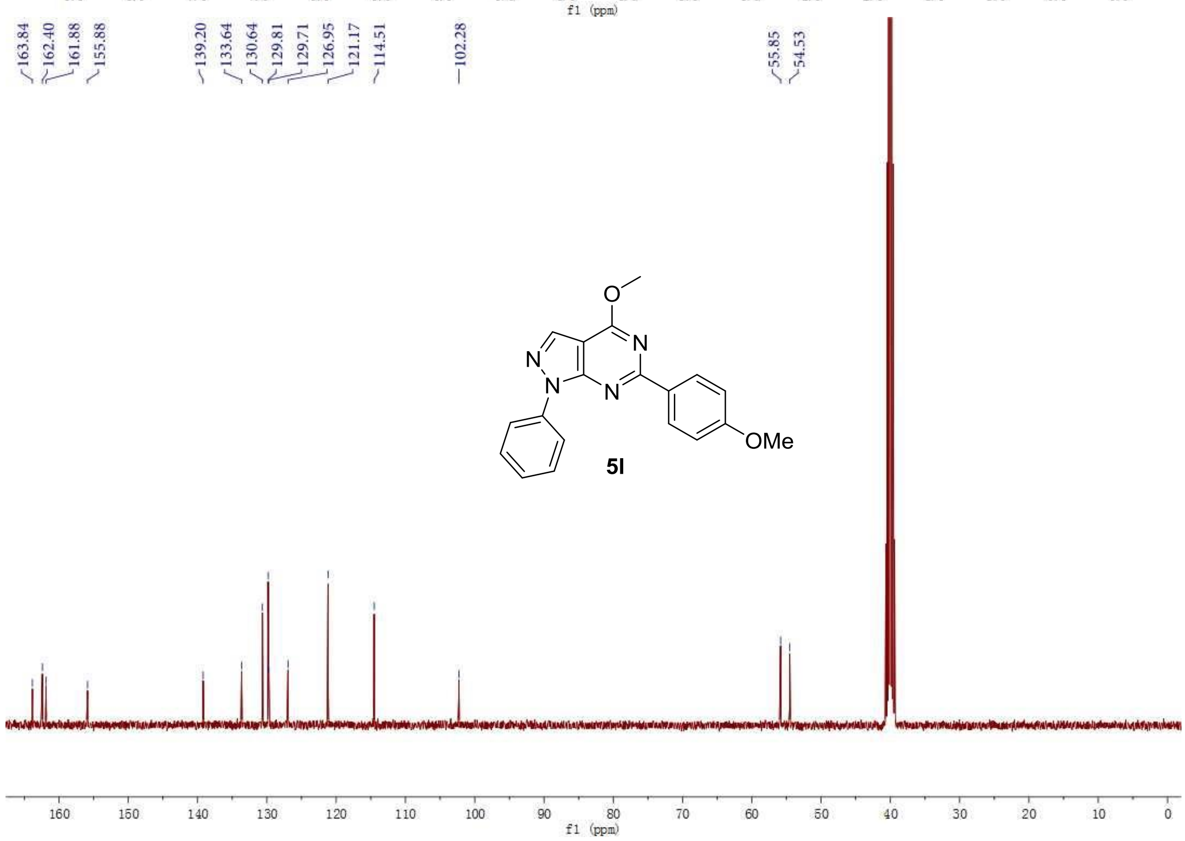
163.84
162.40
161.88
155.88
139.20
133.64
130.64
129.81
129.71
126.95
121.17
114.51
102.28
55.85
54.53
N
N
N
N
O
OMe
5l
160 150 140 130 120 110 100 90 80 70 60 50 40 30 20 10 0
f1 (ppm)

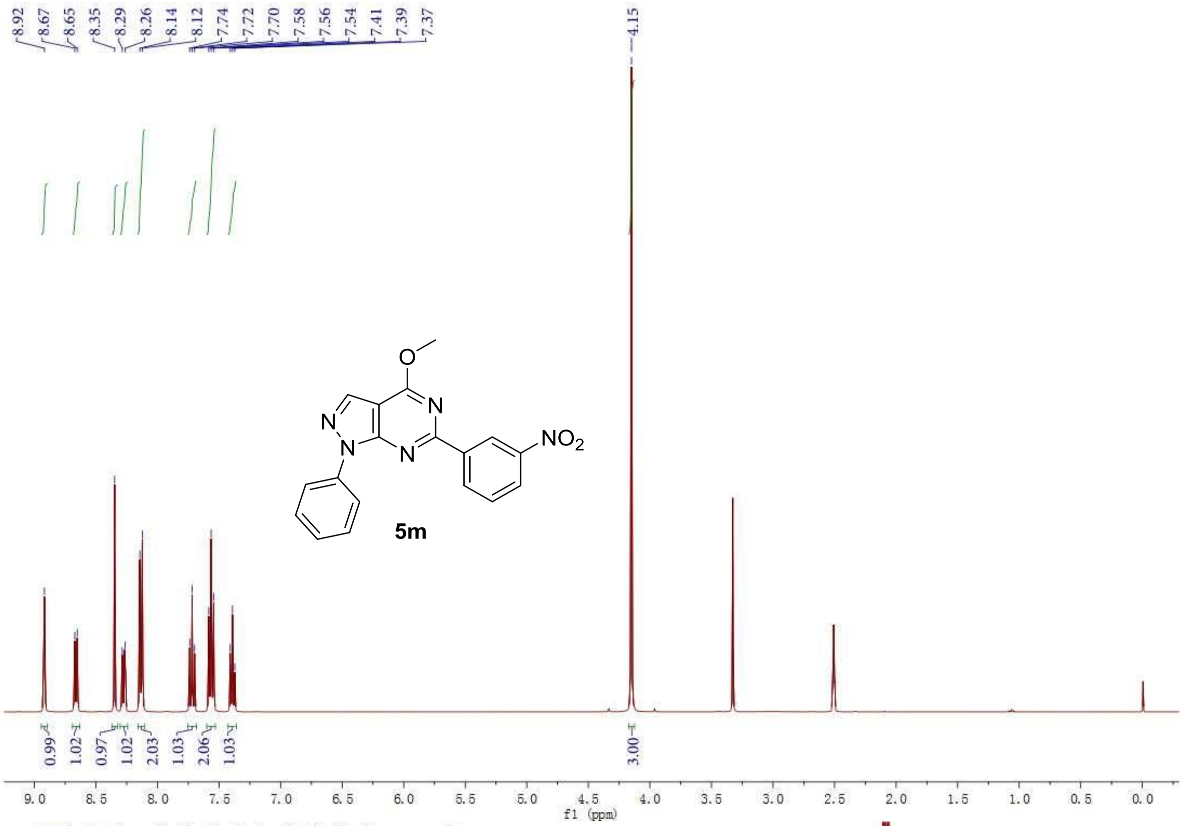
8.92 8.67 8.65 8.35 8.29 8.26 8.14 8.12 7.74 7.72 7.70 7.58 7.56 7.54 7.41 7.39 7.37
4.15
0.99 1.02 0.97 1.02 2.03 1.03 2.06 1.03
3.00
NO2
5m
f1 (ppm)

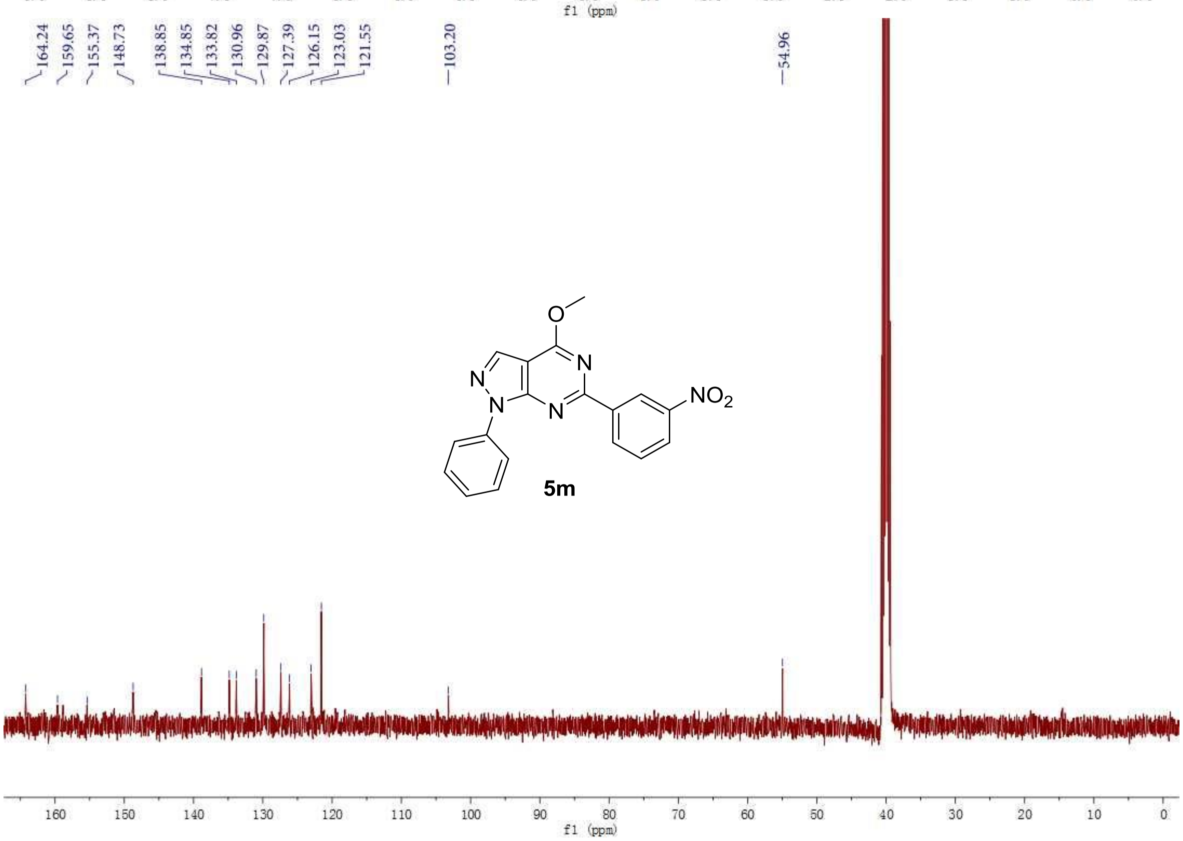
164.24 159.65 155.37 148.73 138.85 134.85 133.82 130.96 129.87 127.39 126.15 123.03 121.55
103.20
54.96
NO2
5m
f1 (ppm)

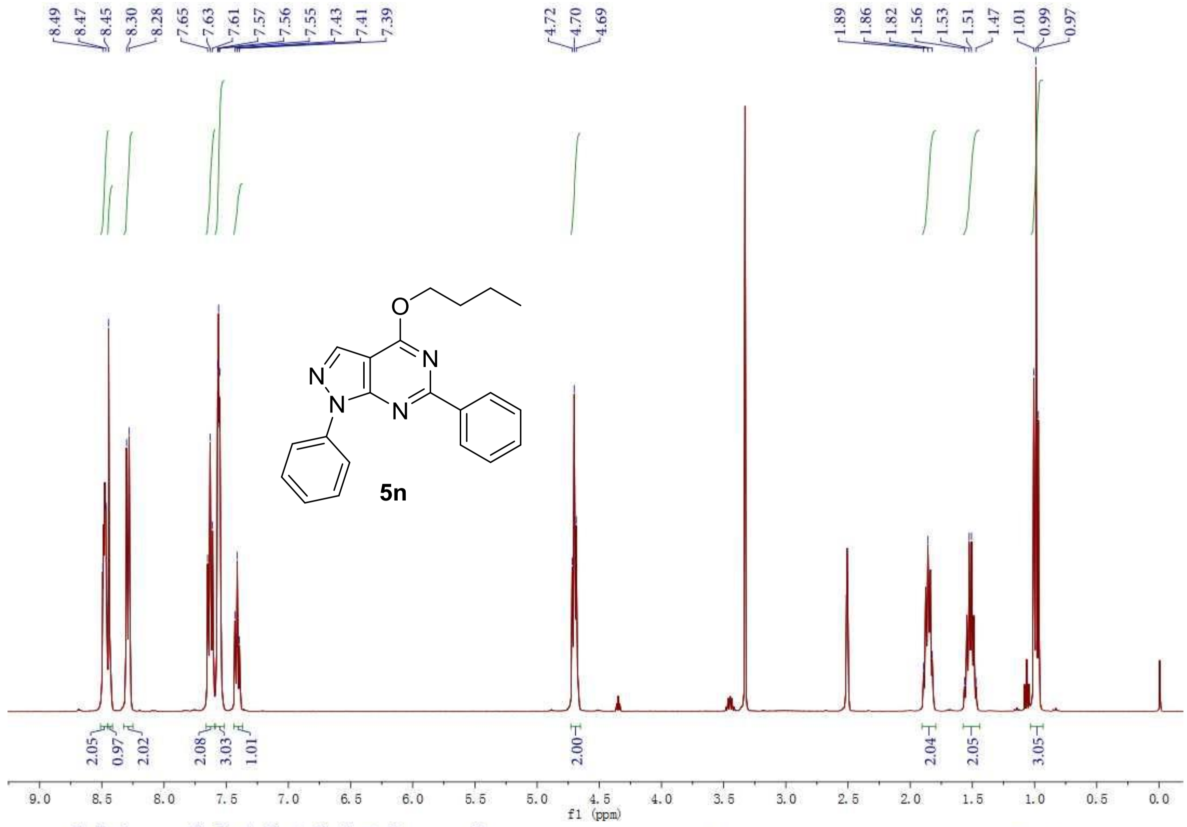
8.49 8.47 8.45 8.30 8.28 7.65 7.63 7.61 7.57 7.56 7.55 7.43 7.41 7.39
4.72 4.70 4.69
1.89 1.86 1.82 1.56 1.53 1.51 1.47 1.01 0.99 0.97
O
N
N
N
N
5n
2.05 0.97 2.02 2.08 3.03 1.01 2.00 2.04 2.05 3.05
f1 (ppm)

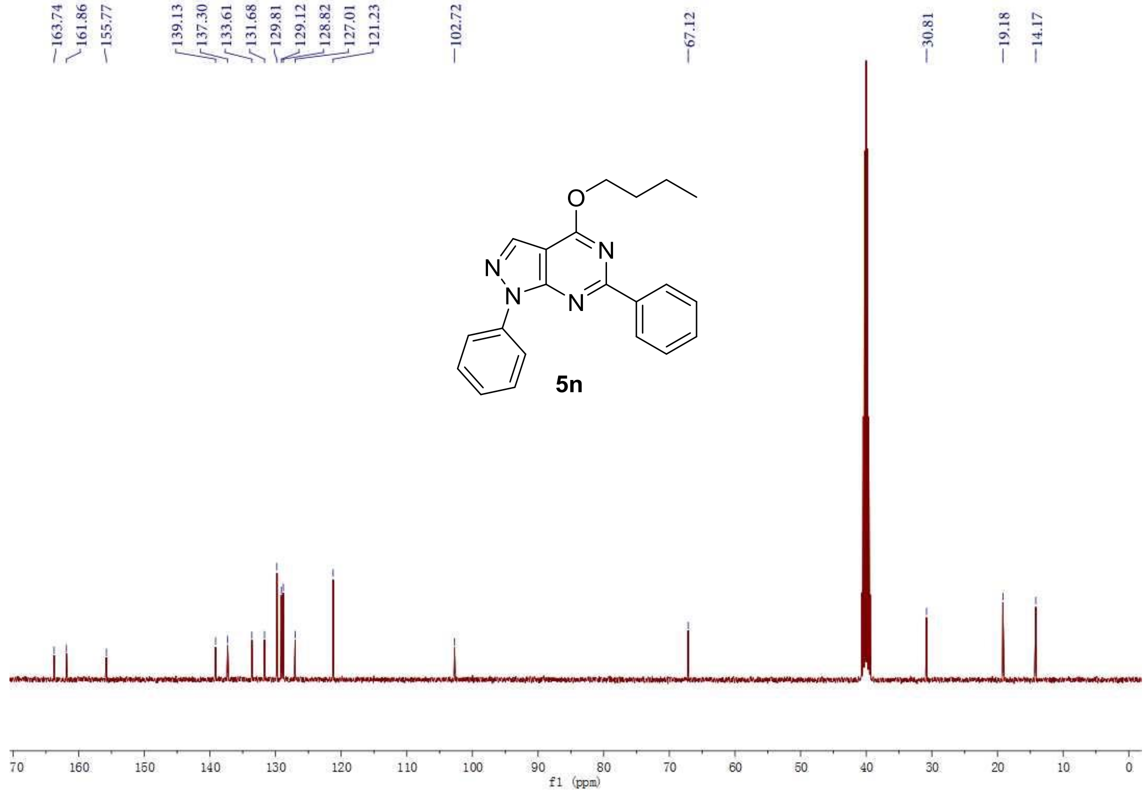
163.74 161.86 155.77 139.13 137.30 133.61 131.68 129.81 129.12 128.82 127.01 121.23 102.72 67.12 30.81 19.18 14.17
O
N
N
N
N
5n
f1 (ppm)

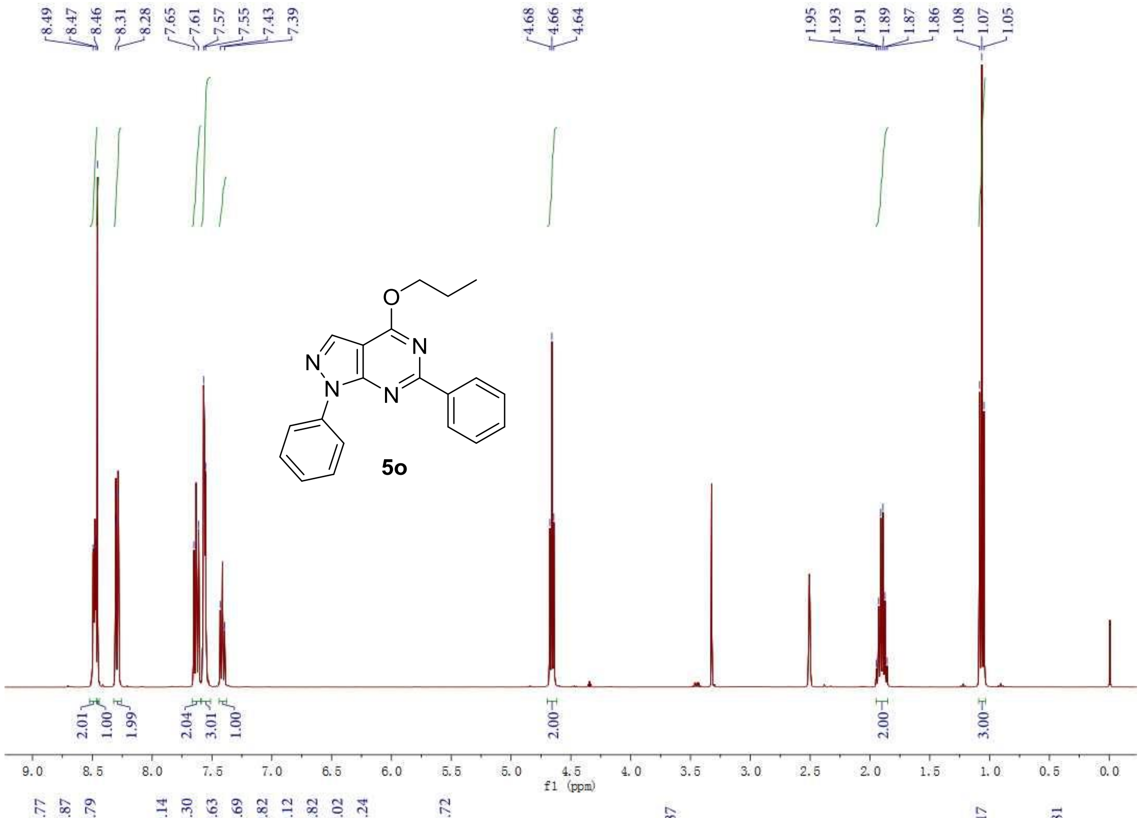
8.49
8.47
8.46
8.31
8.28
7.65
7.61
7.57
7.55
7.43
7.39
4.68
4.66
4.64
1.95
1.93
1.91
1.89
1.87
1.86
1.08
1.07
1.05
N
N
N
N
O
5o
2.01
1.00
1.99
2.04
3.01
1.00
2.00
2.00
3.00
f1 (ppm)

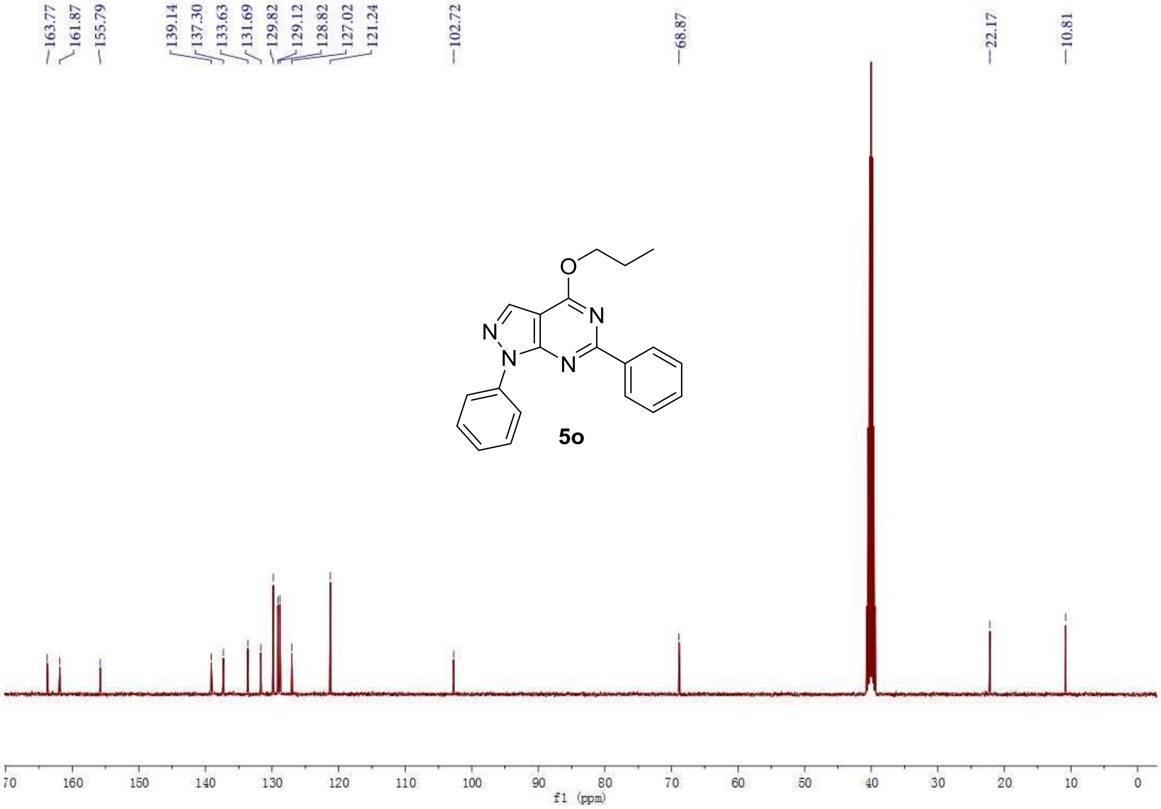
163.77
161.87
155.79
139.14
137.30
133.63
131.69
129.82
129.12
128.82
127.02
121.24
102.72
68.87
22.17
10.81
N
N
N
N
O
5o
f1 (ppm)

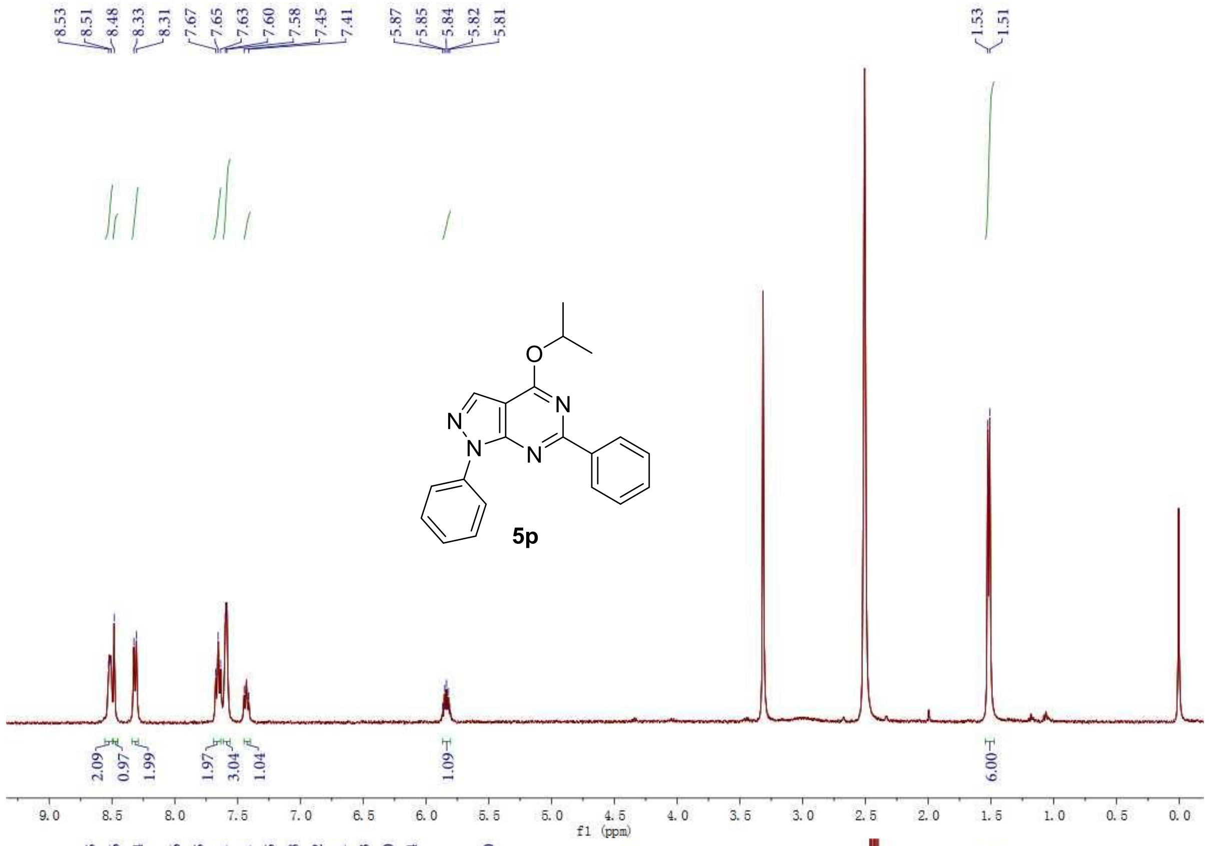

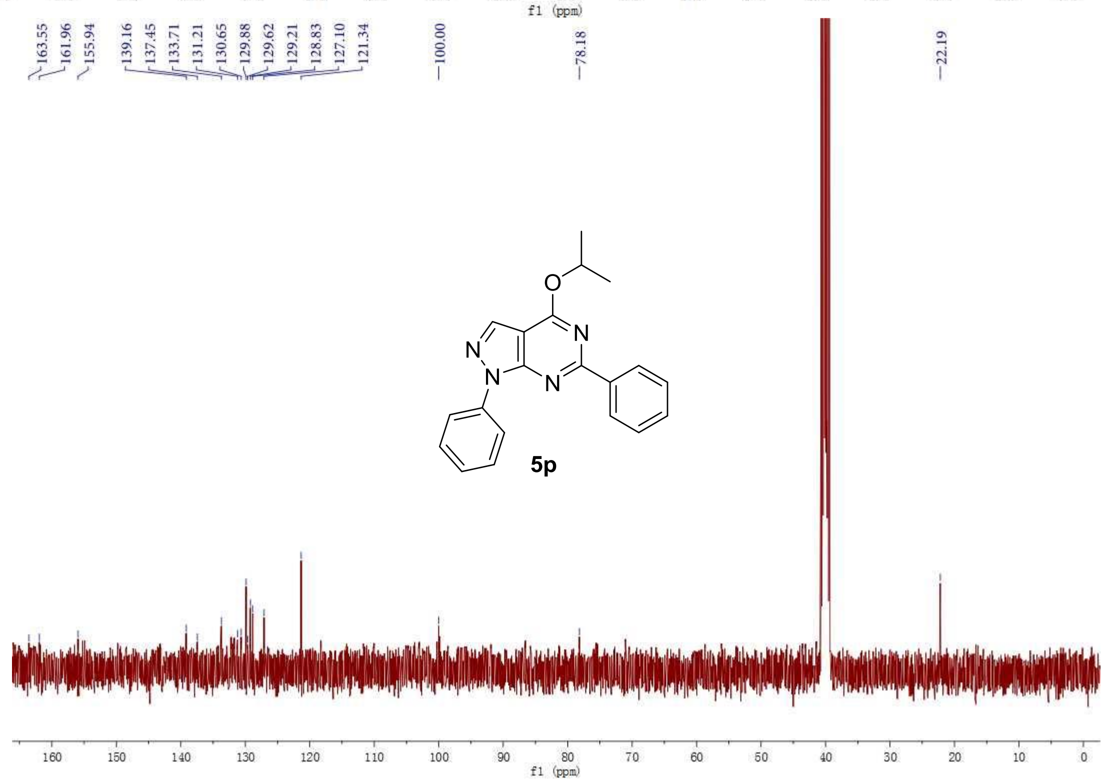

HRMS spectra of 5

Peking University Mass Spectrometry Sample Analysis Report

Analysis Info

Analysis Name	15041135_20150429_000001.d	Acquisition Date	4/29/2015 10:51:49 AM
Sample	lmx-046-1	Instrument	Bruker Apex IV FTMS
Comment	ESI Positive	Operator	Peking University

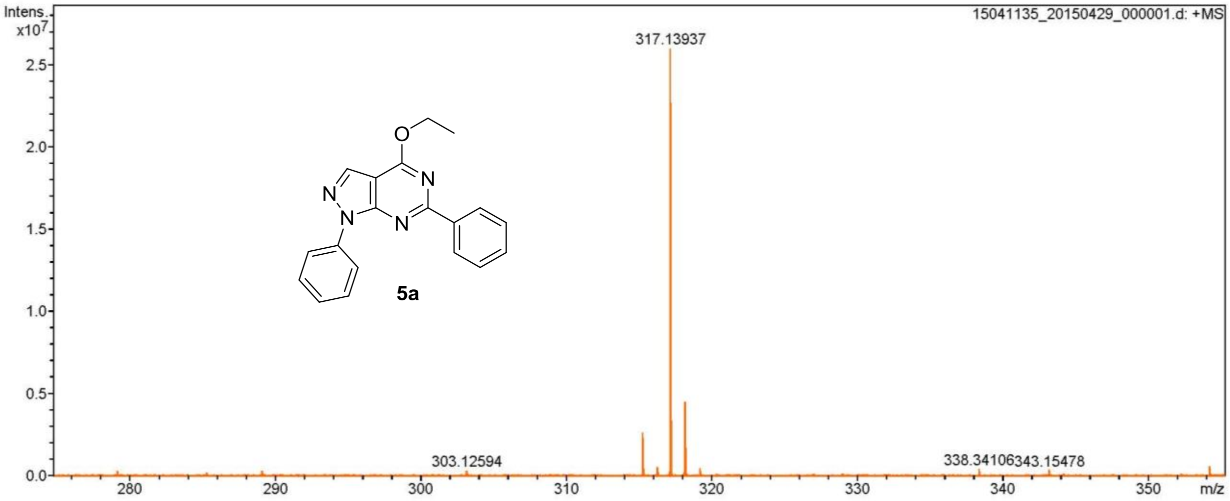

Meas. m/z	I	Formula	Score	m/z	err [mDa]	err [ppm]	mSigma	rdb	e⁻ Conf	N-Rule
317.13937	26033240	C 19 H 17 N 4 O	100.00	317.13969	0.3	1.0	26.0	13.5	even	ok

Peking University Mass Spectrometry Sample Analysis Report

Analysis Info

Analysis Name	15041135_20150429_000002.d	Acquisition Date	4/29/2015 10:55:56 AM
Sample	lmx-033-1	Instrument	Bruker Apex IV FTMS
Comment	ESI Positive	Operator	Peking University

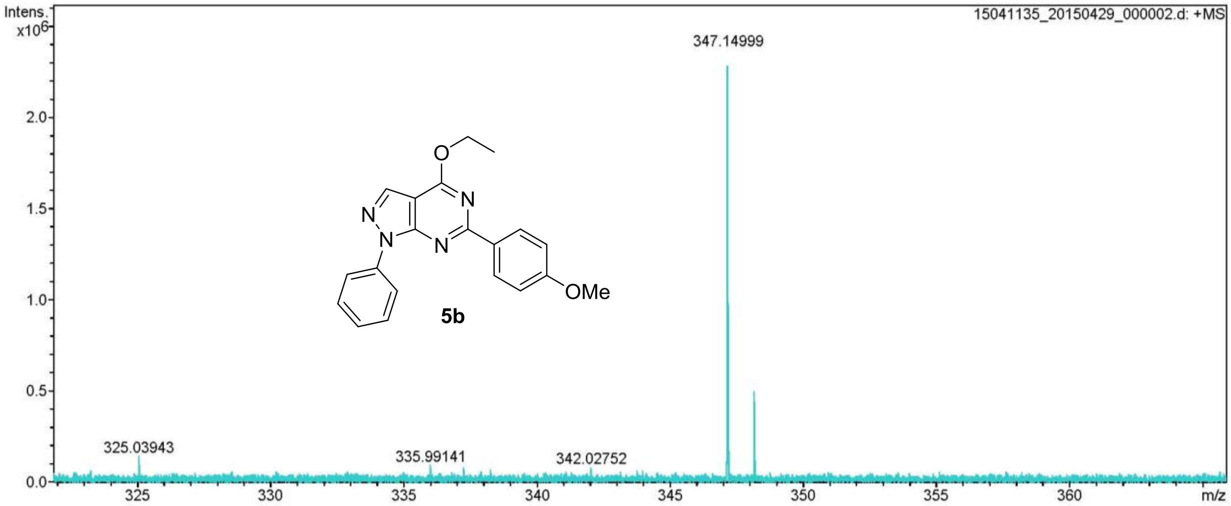

Meas. m/z	I	Formula	Score	m/z	err [mDa]	err [ppm]	mSigma	rdb	e⁻ Conf	N-Rule
347.14999	2369316	C 20 H 19 N 4 O 2	100.00	347.15025	0.3	0.8	19.3	13.5	even	ok

Peking University Mass Spectrometry Sample Analysis Report

Analysis Info

Analysis Name	15050836_20150521_000005.d	Acquisition Date	5/21/2015 11:04:15 AM
Sample	Lmx-052-6	Instrument	Bruker Apex IV FTMS
Comment	ESI Positive	Operator	Peking University

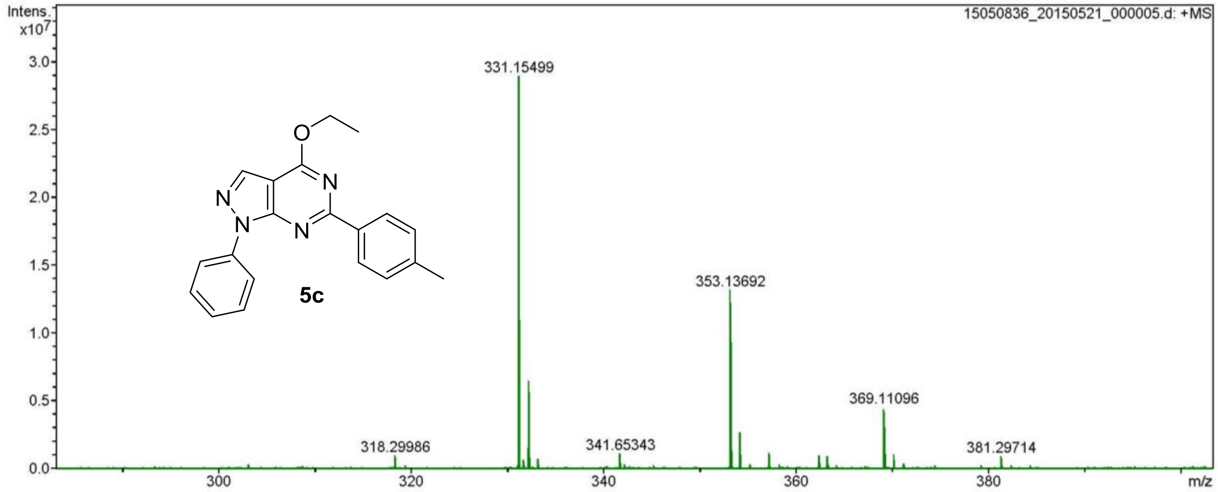

Meas. m/z	I	Formula	Score	m/z	err [mDa]	err [ppm]	mSigma	rdb	e⁻ Conf	N-Rule
331.15499	28942608	C 20 H 19 N 4 O	100.00	331.15534	0.4	1.1	3.2	13.5	even	ok

Peking University Mass Spectrometry Sample Analysis Report

Analysis Info

Analysis Name	15050836_20150521_000006.d	Acquisition Date	5/21/2015 11:05:49 AM
Sample	Lmx-052-4	Instrument	Bruker Apex IV FTMS
Comment	ESI Positive	Operator	Peking University

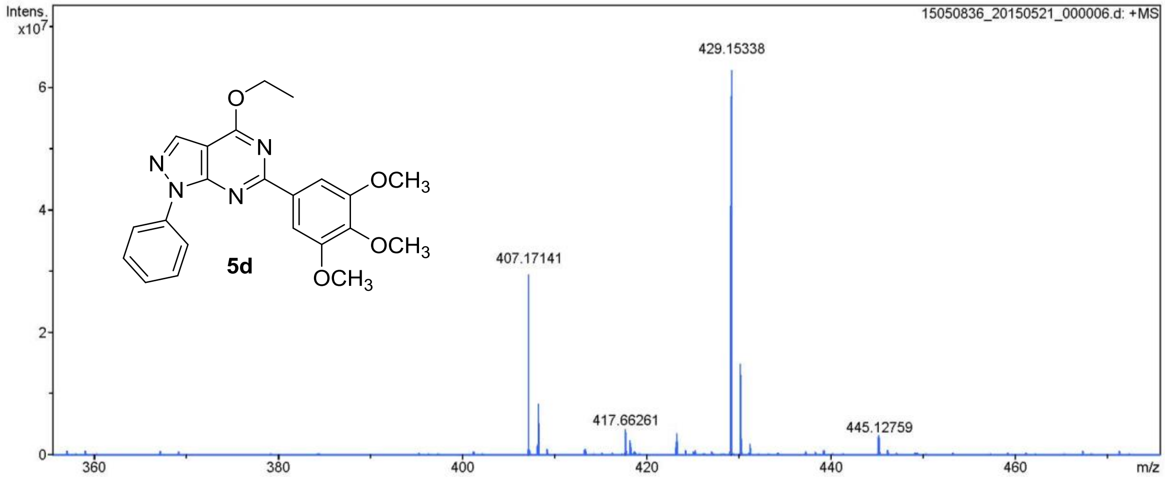

Meas. m/z	I	Formula	Score	m/z	err [mDa]	err [ppm]	mSigma	rdb	e⁻ Conf	N-Rule
407.17141	30737866	C 22 H 23 N 4 O 4	100.00	407.17138	-0.0	-0.1	10.9	13.5	even	ok

Peking University Mass Spectrometry Sample Analysis Report

Analysis Info

Analysis Name	15050836_20150521_000004.d	Acquisition Date	5/21/2015 11:00:44 AM
Sample	Lmx-052-5	Instrument	Bruker Apex IV FTMS
Comment	ESI Positive	Operator	Peking University

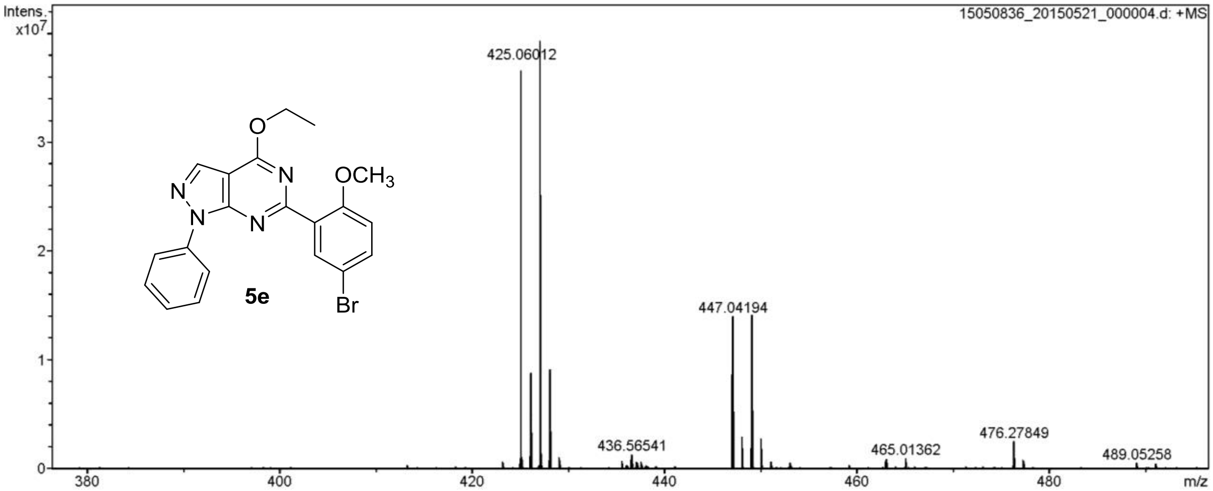

Meas. m/z	I	Formula	Score	m/z	err [mDa]	err [ppm]	mSigma	rdb	e⁻ Conf	N-Rule
425.06012	37261440	C 20 H 18 Br N 4 O 2	100.00	425.06076	0.6	1.5	447.3	13.5	even	ok

Peking University Mass Spectrometry Sample Analysis Report

Analysis Info

Analysis Name	15041135_20150429_000009.d	Acquisition Date	4/29/2015 11:33:49 AM
Sample	lmx-050-1	Instrument	Bruker Apex IV FTMS
Comment	ESI Positive	Operator	Peking University

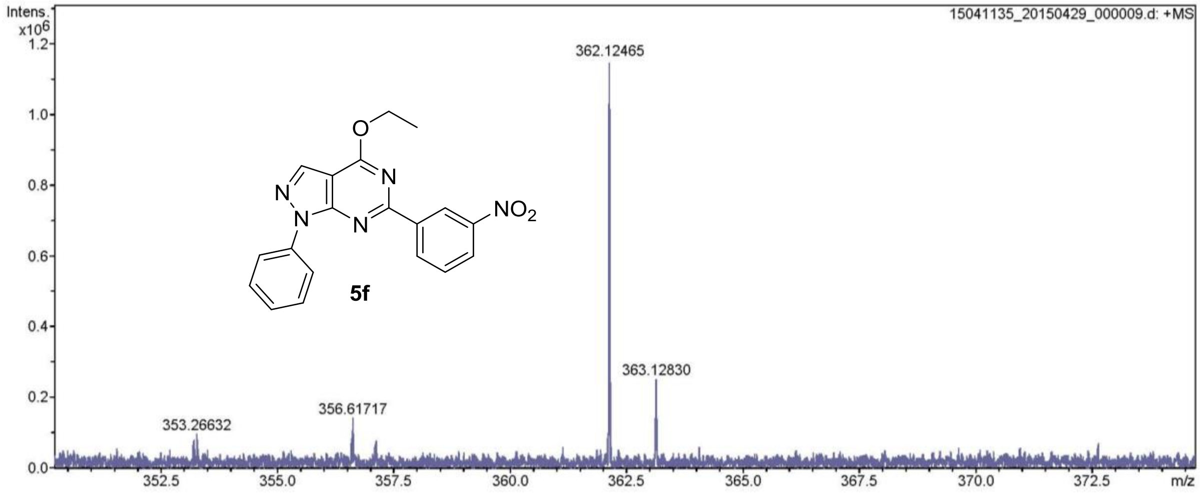

Meas. m/z	I	Formula	Score	m/z	err [mDa]	err [ppm]	mSigma	rdb	e⁻ Conf	N-Rule
362.12465	1156214	C 19 H 16 N 5 O 3	100.00	362.12477	0.1	0.3	17.6	14.5	even	ok

Peking University Mass Spectrometry Sample Analysis Report

Analysis Info

Analysis Name	15050588_20150515_000006.d	Acquisition Date	5/15/2015 9:46:53 AM
Sample	lmx-05[illegible]5	Instrument	Bruker Apex IV FTMS
Comment	ESI Positive	Operator	Peking University

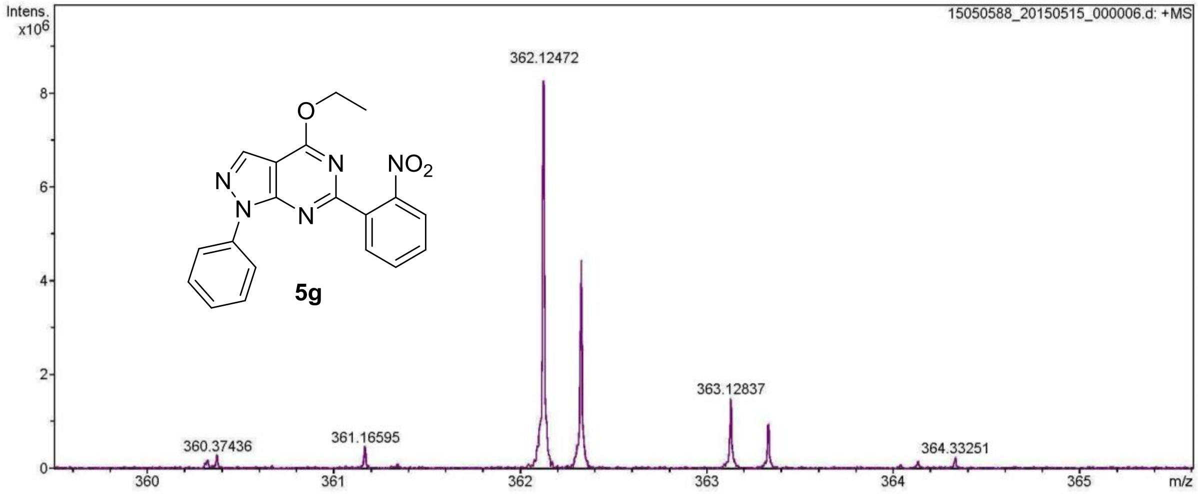

Meas. m/z	I	Formula	Score	m/z	err [mDa]	err [ppm]	mSigma	rdb	e⁻ Conf	N-Rule
362.12472	8571883	C 19 H 16 N 5 O 3	100.00	362.12477	0.0	0.1	28.4	14.5	even	ok

Peking University Mass Spectrometry Sample Analysis Report

Analysis Info

Analysis Name	15050588_20150515_000005.d	Acquisition Date	5/15/2015 9:45:21 AM
Sample	lmx-051-2	Instrument	Bruker Apex IV FTMS
Comment	ESI Positive	Operator	Peking University

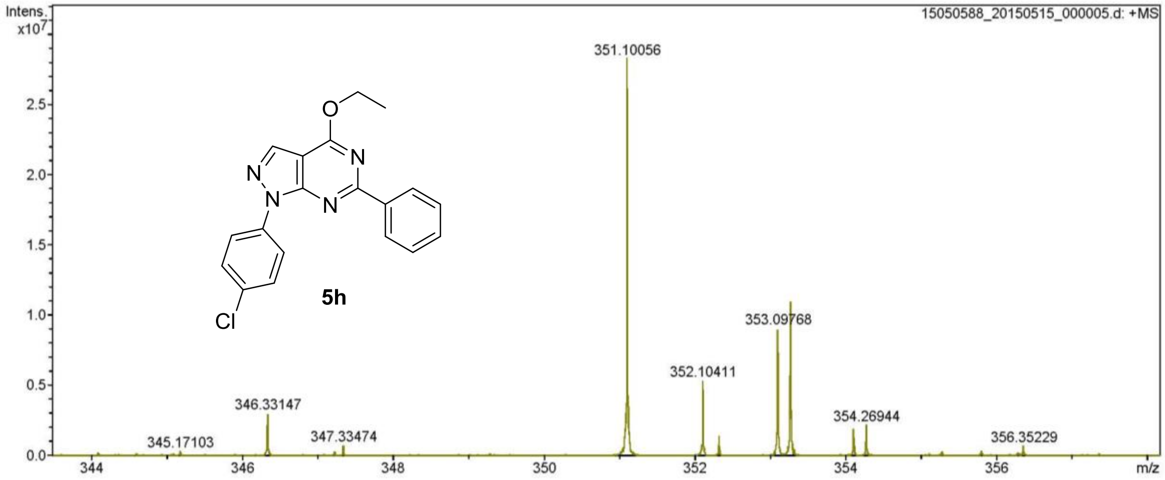

Meas. m/z	I	Formula	Score	m/z	err [mDa]	err [ppm]	mSigma	rdb	e⁻ Conf	N-Rule
351.10056	28309086	C 19 H 16 Cl N 4 O	100.00	351.10072	0.2	0.4	16.9	13.5	even	ok

Peking University Mass Spectrometry Sample Analysis Report

Analysis Info

Analysis Name	15050588_20150515_000004.d	Acquisition Date	5/15/2015 9:43:49 AM
Sample	lmx-051-1	Instrument	Bruker Apex IV FTMS
Comment	ESI Positive	Operator	Peking University

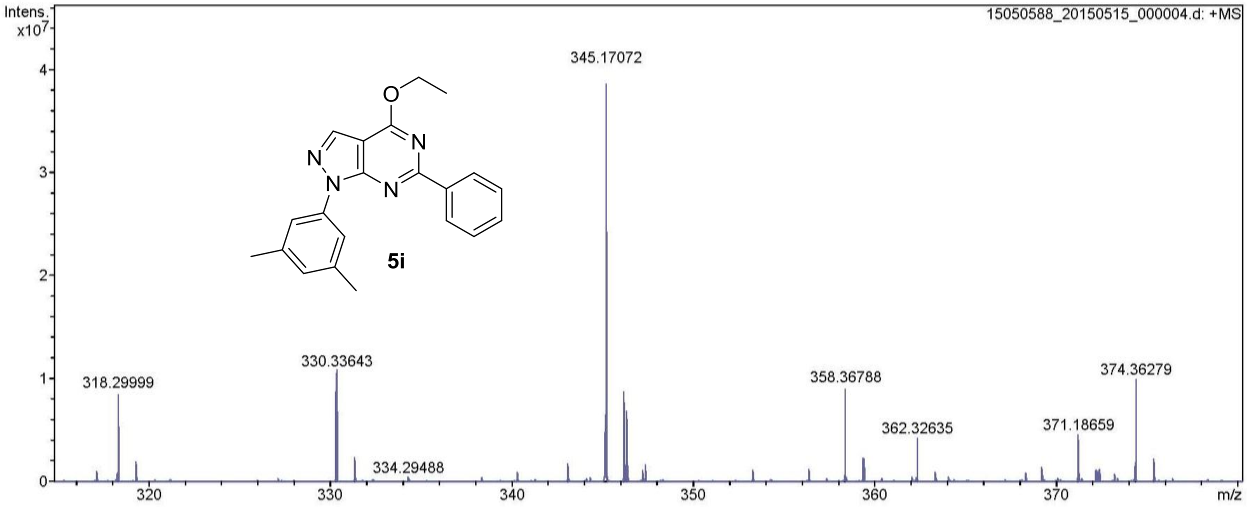

Meas. m/z	I	Formula	Score	m/z	err [mDa]	err [ppm]	mSigma	rdb	e⁻ Conf	N-Rule
345.17072	40325784	C 21 H 21 N 4 O	100.00	345.17099	0.3	0.8	15.2	13.5	even	ok

Peking University Mass Spectrometry Sample Analysis Report

Analysis Info

Analysis Name	15041135_20150429_000006.d	Acquisition Date	4/29/2015 11:17:29 AM
Sample	lmx-050-4	Instrument	Bruker Apex IV FTMS
Comment	ESI Positive	Operator	Peking University

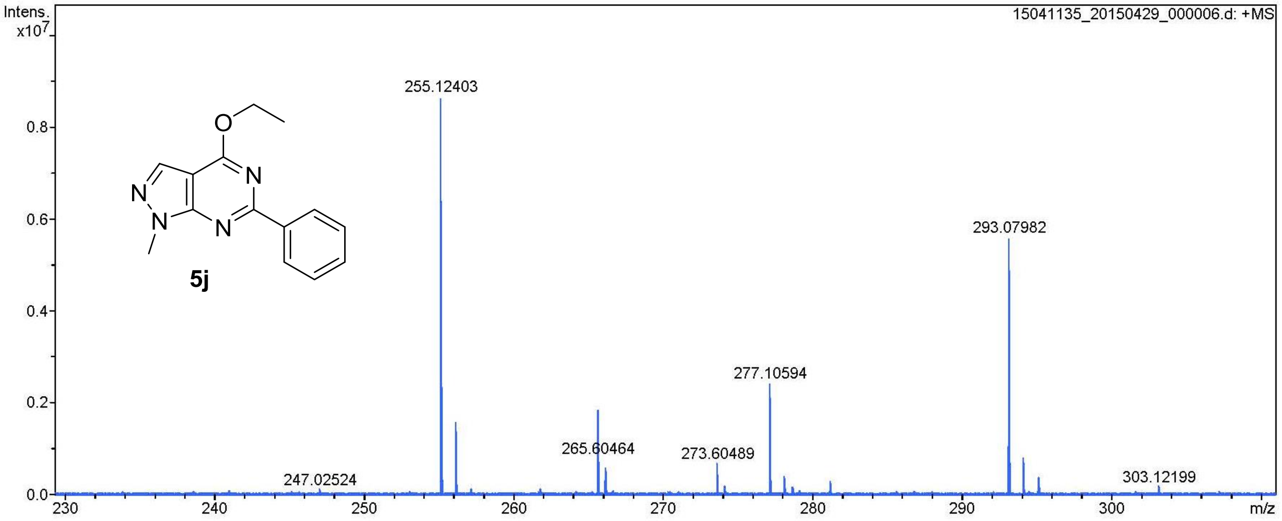

Meas. m/z	I	Formula	Score	m/z	err [mDa]	err [ppm]	mSigma	rdb	e⁻ Conf	N-Rule
255.12403	8694393	C 14 H 15 N 4 O	100.00	255.12404	0.0	0.0	9.4	9.5	even	ok

Peking University Mass Spectrometry Sample Analysis Report

Analysis Info

Analysis Name	15050588_20150515_000007.d	Acquisition Date	5/15/2015 9:49:26 AM
Sample	lmx-050-7	Instrument	Bruker Apex IV FTMS
Comment	ESI Positive	Operator	Peking University

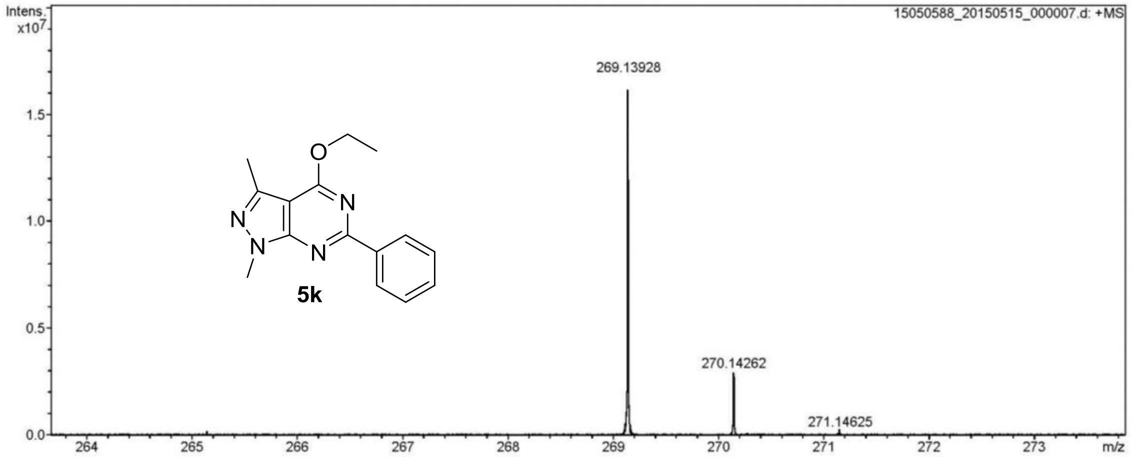

Meas. m/z	I	Formula	Score	m/z	err [mDa]	err [ppm]	mSigma	rdb	e^- Conf	N-Rule
269.13928	16817298	C 15 H 17 N 4 O	100.00	269.13969	0.4	1.5	0.3	9.5	even	ok

Peking University Mass Spectrometry Sample Analysis Report

Analysis Info

Analysis Name	15041135_20150429_000005.d	Acquisition Date	4/29/2015 11:15:25 AM
Sample	lmx-050-2	Instrument	Bruker Apex IV FTMS
Comment	ESI Positive	Operator	Peking University

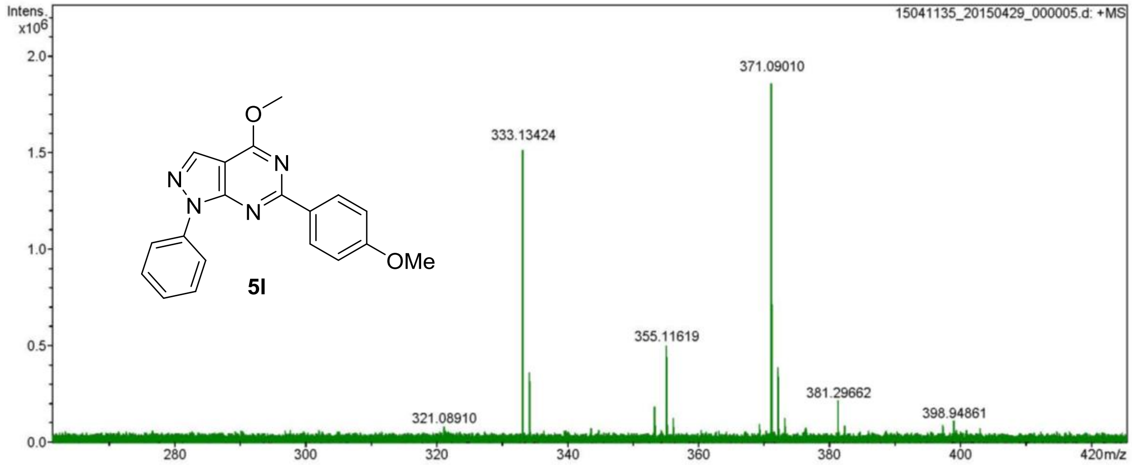

Meas. m/z	I	Formula	Score	m/z	err [mDa]	err [ppm]	mSigma	rdb	e^- Conf	N-Rule
333.13424	1551799	C 19 H 17 N 4 O 2	100.00	333.13460	0.4	1.1	17.6	13.5	even	ok

Peking University Mass Spectrometry Sample Analysis Report

Analysis Info

Analysis Name	15041135_20150429_000008.d	Acquisition Date	4/29/2015 11:31:45 AM
Sample	lmx-050-3	Instrument	Bruker Apex IV FTMS
Comment	ESI Positive	Operator	Peking University

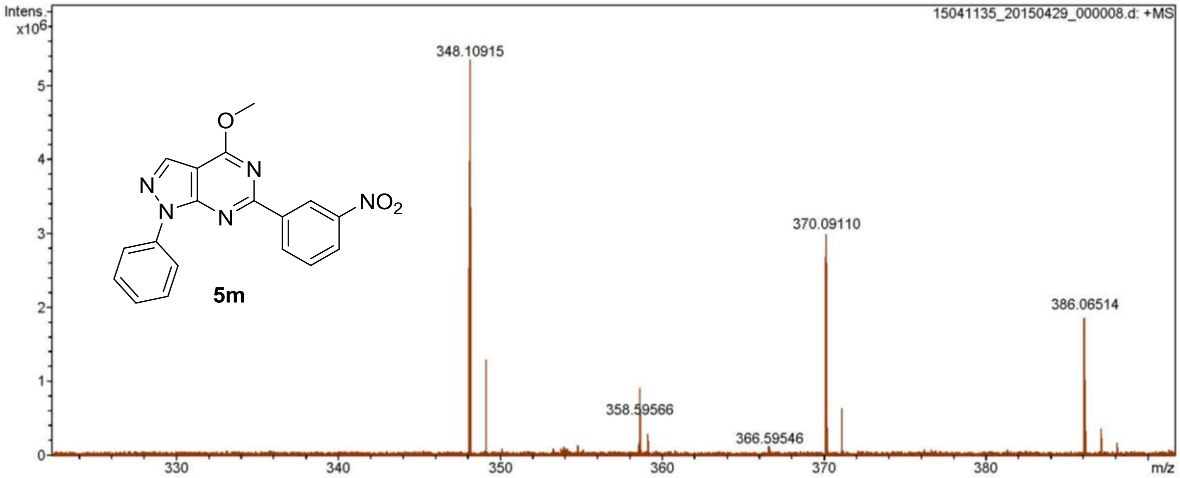

Meas. m/z	I	Formula	Score	m/z	err [mDa]	err [ppm]	mSigma	rdb	e⁻ Conf	N-Rule
348.10915	5349086	C 18 H 14 N 5 O 3	100.00	348.10912	-0.0	-0.1	16.5	14.5	even	ok

Peking University Mass Spectrometry Sample Analysis Report

Analysis Info

Analysis Name	15050836_20150521_000001.d	Acquisition Date	5/21/2015 10:09:50 AM
Sample	Lmx-052-3	Instrument	Bruker Apex IV FTMS
Comment	ESI Positive	Operator	Peking University

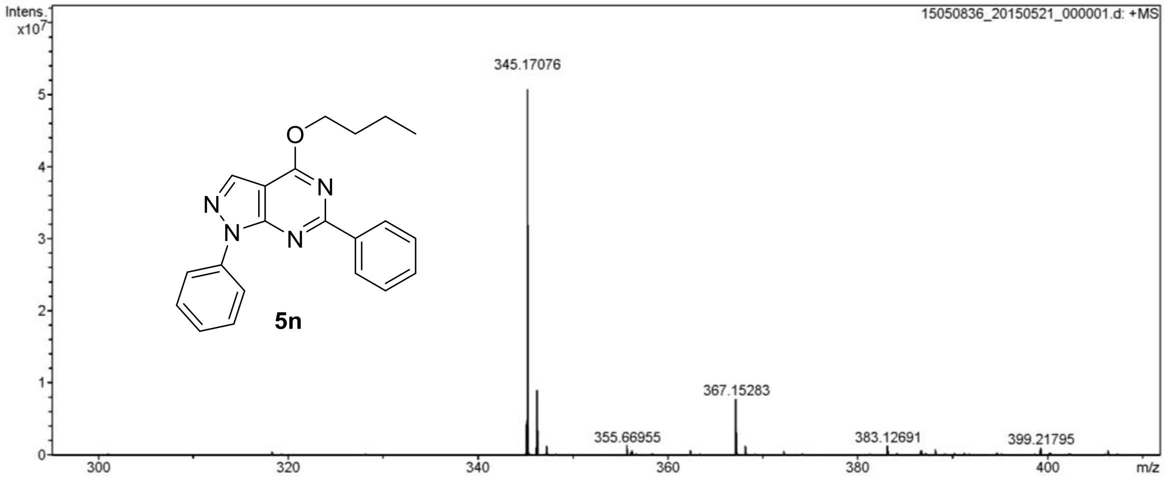

Meas. m/z	I	Formula	Score	m/z	err [mDa]	err [ppm]	mSigma	rdb	e⁻ Conf	N-Rule
345.17076	52961308	C 21 H 21 N 4 O	100.00	345.17099	0.2	0.6	41.9	13.5	even	ok

Peking University Mass Spectrometry Sample Analysis Report

Analysis Info

Analysis Name	15050836_20150521_000002.d	Acquisition Date	5/21/2015 10:57:37 AM
Sample	Lmx-052-1	Instrument	Bruker Apex IV FTMS
Comment	ESI Positive	Operator	Peking University

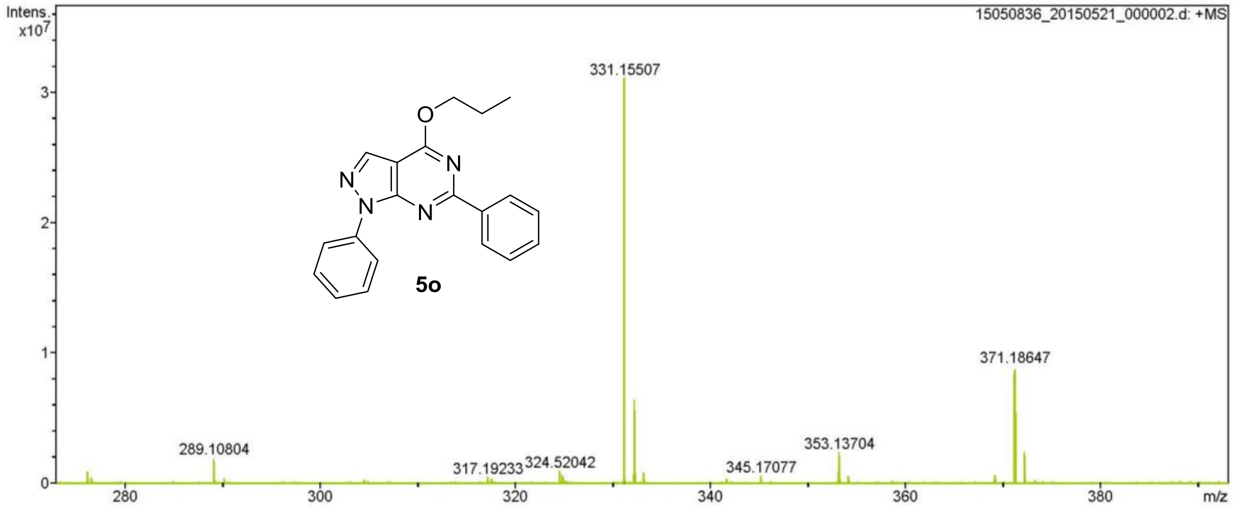

Meas. m/z	I	Formula	Score	m/z	err [mDa]	err [ppm]	mSigma	rdb	e⁻ Conf	N-Rule
331.15507	31068354	C 20 H 19 N 4 O	100.00	331.15534	0.3	0.8	12.2	13.5	even	ok

Peking University Mass Spectrometry Sample Analysis Report

Analysis Info

Analysis Name	15050836_20150521_000003.d	Acquisition Date	5/21/2015 10:59:17 AM
Sample	Lmx-052-2	Instrument	Bruker Apex IV FTMS
Comment	ESI Positive	Operator	Peking University

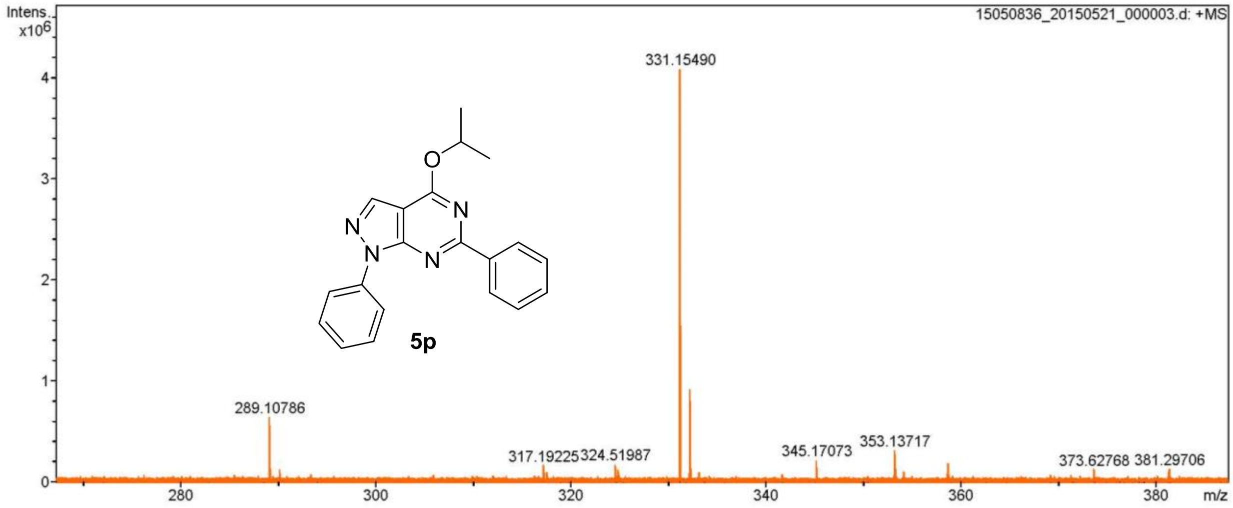

Meas. m/z	I	Formula	Score	m/z	err [mDa]	err [ppm]	mSigma	rdb	e⁻ Conf	N-Rule
331.15490	4085502	C 20 H 19 N 4 O	100.00	331.15534	0.4	1.3	3.0	13.5	even	ok
